# Genome-wide analysis of the MADS-box gene family of sea buckthorn (*Hippophae rhamnoides ssp. sinensis*) and their potential role in floral organ development

**DOI:** 10.3389/fpls.2024.1387613

**Published:** 2024-06-13

**Authors:** Jing Zhao, Yazhuo Xu, Zhihua Zhang, Meng Zhao, Kai Li, Fanhong Wang, Kun Sun

**Affiliations:** College of Life Science, Northwest Normal University, Lanzhou, China

**Keywords:** MADS-box gene family, sea buckthorn, floral organ development, expression patterns, sex differentiation

## Abstract

Sea buckthorn (*Hippophae rhamnoides* ssp. *sinensis*) is a deciduous shrub or small tree in the Elaeagnaceae family. It is dioecious, featuring distinct structures in female and male flowers. The MADS-box gene family plays a crucial role in flower development and differentiation of floral organs in plants. However, systematic information on the MADS-box family in sea buckthorn is currently lacking. This study presents a genome-wide survey and expression profile of the MADS-box family of sea buckthorn. We identified 92 MADS-box genes in the *H. rhamnoides* ssp. *Sinensis* genome. These genes are distributed across 12 chromosomes and classified into Type I (42 genes) and Type II (50 genes). Based on the FPKM values in the transcriptome data, the expression profiles of HrMADS genes in male and female flowers of sea buckthorn showed that most Type II genes had higher expression levels than Type I genes. This suggesting that Type II *HrMADS* may play a more significant role in sea buckthorn flower development. Using the phylogenetic relationship between sea buckthorn and *Arabidopsis thaliana*, the ABCDE model genes of sea buckthorn were identified and some ABCDE model-related genes were selected for qRT-PCR analysis in sea buckthorn flowers and floral organs. Four B-type genes may be involved in the identity determination of floral organs in male flowers, and D-type genes may be involved in pistil development. It is hypothesized that ABCDE model genes may play an important role in the identity of sea buckthorn floral organs. This study analyzed the role of MADS-box gene family in the development of flower organs in sea buckthorn, which provides an important theoretical basis for understanding the regulatory mechanism of sex differentiation in sea buckthorn.

## 1 Introduction

The family of MADS-box genes is a set of transcription factors found across eukaryotes that play a crucial role in controlling various aspects of plant growth and development. These include the formation of flower parts, development of male and female reproductive cells, growth of embryos, seeds, and fruits, in addition to functions like photosynthesis, nutrient processing, differentiation of apical meristems, and signaling of hormones (Becker and Theissen, 2003; Messenguy and Dubois, 2003). MADS structural domain proteins are characterized by a MADS structural domain consisting of 56–58 amino acids and are named after the initial letters of the four earliest discovered MADS-box genes: *MINICHROMOSOME MAINTENANCE1* in yeast, *AGAMOUS* in *Arabidopsis*, *DEFICIENS* in *Antirrhinum majus*, and *SERUM RESPONSE FACTOR* in humans (Alvarez-Buylla et al., 2000; Gramzow and Theissen, 2010). The MADS domain recognizes a CArG-box with a similar 10 bp A/T-rich DNA sequence (Folter and Angenent, 2006). Based on gene structure and phylogenetic relationships, plant MADS-box family genes are categorized into Type I (SRF-like) and Type II (which can be referred to as MEF2-like or MIKC-type) (Becker and Theissen, 2003). The Type II MADS-box gene contains four structural domains: the MADS domain (MADS-box), the I region (Intervening), the K domain (Keratin-like) and the C-terminal (C-terminal) (Münster et al., 1997), whereas Type I MADS-box genes lack the K structural domain, MIKC types can be further classified into MIKC* and MIKC^C^ types based on the sequence length of the K domain and the I region (Parenicová et al., 2003). The maximum likelihood evolutionary tree of all MIKC^C^-type MADS-box genes in *Arabidopsis thaliana*, rice (*Oryza sativa*), and wheat classifies the MIKC^C^-type MADS-box genes into 12 subfamilies (Gramzow and Theissen, 2015). Type I is subdivided into Mα, Mβ, and Mγ subgroups (Kofuji et al., 2003). MIKC^C^-type proteins are currently the most well-studied functional group within the MADS-box gene family in plants, in contrast to Type I MADS-boxand MIKC*-type proteins, which have been less reported. However, a few studies have demonstrated that these less-studied types play an important role in the development of male and female gametophytes in plants (Adamczyk and Fernandez, 2009; Masiero et al., 2011).

Flowering is an intricate biological process that requires the coordination and interaction of many genes. Using *Arabidopsis* and snapdragon as examples, scientists have started to reveal the genetic regulatory pathways of plant floral organ formation and have put forward the famous ABCDE model of floral organ development (Coen and Meyerowitz, 1991; Agrawal et al., 2005). Flowers typically consist of four whorls of organs arranged from the outside to the inside, in the order of sepals, petals, stamens and carpels. With the exception of AP2, all genes participating in the ABCDE model are MADS-box genes from various functional categories (Gramzow and Theissen, 2010). The ABCDE model provides a comprehensive explanation of the developmental processes of plant flowers and the specification of floral organ identities (Ma, 2000; Theissen et al., 2000). In *Arabidopsis*, class A genes (*APETALA1*) control the development of sepals and petals; class B genes (*PISTILLATA*, *APETALA3*) control the development of petals and stamens; class C genes (*AGAMOUS*) control the development of stamens and carpels; class D genes (*SEEDSTICK, SHATTERPROOF*) control the development of the ovule; and class E genes (*SEPALLATA 1, 2, 3, 4*) are involved in the development of all floral organs (Ma, 2000). Additionally, a tetrameric protein complex comprising the ABCDE genes determines the identity of the floral organ during development (Theißen, 2001).

Sea buckthorn is a deciduous shrub or small tree in the Elaeagnaceae family, recognized for its economic importance and soil conservation value. All sea buckthorn plants are dioecious. They flower from April to May, with flowers opening before the leaves appear. The flowers are small, lack corollas, and are considered incomplete (Zhang, 2006). Male flowers consist of 2 bracts, 2 sepals, and 4 stamens, while female flowers comprise 2 bracts and a pistil enclosed by perianth tube. The mechanism determining their sexual differentiation remains unclear. 21 genes were isolated and characterized in sea buckthorn that were differentially expressed at the male or female bud stage, of which *HrCRY2* was significantly expressed only in female buds, while *HrCO* was significantly expressed only in male flowers (Chawla et al., 2015). The information of these sex-specific expressed genes will help to elucidate the mechanism of sex determination in sea buckthorn, however, the mechanism of its sex differentiation decision is still unknown. Recent research has suggested that MADS-box genes may play a significant role in plant sex differentiation. For instance, in the study of ABCDE model-related genes in the flower sex differentiation of kiwifruit (*Actinidia chinensis* Planch.), it was found that *AcMADS4*, *AcMADS56*, and *AcMADS70* may be involved in differentiating male and female flowers in kiwifruit, may be further involved in sex differentiation (Ye et al., 2022). Similarly, ABCDE model-related genes may also play a role in regulating the differentiation of male and female flowers in sea buckthorn.

This study aims to investigate the functions of MADS-box genes in sea buckthorn and to identify candidate genes that determine the development of its floral organs. Based on the sea buckthorn genome, we identified *HrMADS* genes in sea buckthorn and analyzed their chromosomal localization, gene structure, promoter cis-acting elements, protein conserved motifs, and expression profiles in male and female flowers. Additionally, genes related to the ABCDE model in sea buckthorn were examined and qRT-PCR validation. The results suggest that *HrMADS42* and *HrMADS62* may be involved in stamen identity decision in male flowers, and *HrMADS69* may play an important role in floral organ identity decision in female flowers. These results can provide theoretical and technical references for a better understanding of MADS-box genes.

## 2 Materials and methods

### 2.1 Identification of *HrMADS* genes

All the protein sequences of *H. rhamnoides* ssp*. sinensis*, *A. thaliana*, *Oryza sativa* and *Solanum lycopersicum* were obtained from public databases [*H. rhamnoides* ssp*. sinensis* from CNGBdb (https://db.cngb.org/) (Wu et al., 2022), *A. thaliana* from TAIR (http://www.arabidopsis.org/) databases (Garcia-Hernandez et al., 2002), *Oryza sativa* from RGAP (http://rice.uga.edu/) databases (Kawahara et al., 2013) and *Solanum lycopersicum* from SGN (https://solgenomics.net/) (Fernandez-Pozo et al., 2015). Two strategies were used to identify the MADS-box transcription factor family, first, the gene sequences of *Arabidopsis*, rice and tomato were selected, and the genomes of sea buckthorn were searched by local BLAST using the software TBtools. In addition, from the Pfam protein database (http://pfam.xfam.org) (Mistry et al., 2021), we retrieved the HMM file corresponding to the MADS domain (PF00319) and utilized HMMER3.0 to identify MADS-box genes in the sea buckthorn genome database. We specifically chose e-values below 0.01 and adhered to the default parameters for our search. Subsequently, these proteins were submitted to the SMART (http://smart.embl-heidelberg.de/) (Letunic and Bork, 2018) and the NCBI Batch CD-search (http://www.ncbi.nlm.nih.gov/Structure/cdd/wrpsb.cgi) (Marchler-Bauer et al., 2015) to confirm the presence and completeness of the MADS domain.The MADS proteins were analyzed for sequence length, molecular weight, and isoelectric point (PI) using the compute pI/Mw tool on the ExPASy server (http://web.expasy.org/protparam/) (Wilkins et al., 1999). The subcellular localization of the identified MADS proteins was predicted by the WolfPsort (https://wolfpsort.hgc.jp/) (Horton et al., 2007). In addition, secondary structure prediction of all *HrMADS* proteins was done by the online Website SOPMA (https://npsa-prabi.ibcp.fr/cgibin/npsa_automat.pl?page=/NPSA/npsa_sopma.html) (Combet et al., 2000).

### 2.2 Phylogenetic tree construction and classification of *HrMADS* genes

In order to examine the phylogenetic connections and classification of *HrMADS* genes, the alignment of protein sequences from *HrMADS* genes was carried out through the MUSCLE program within MEGA 11 (Tamura et al., 2021) with standard settings. The NJ(neighbor-joining) phylogenetic tree was created utilizing MEGA 11, implementing the Poisson model and pairwise deletion, and running bootstrap analysis with 1000 replicates. *Arabidopsis* MADS-box genes (*AtMADS*) were used to assist classification (Theissen et al., 2000).The phylogenetic tree were landscaped by iTOL (https://itol.embl.de/) (Letunic and Bork, 2021) and Adobe Illustrator tool (McLean, 2002).

### 2.3 Gene structure, protein conserved motif analysis, and prediction of functional interacting networks

Coding sequences (CDSs) and genome sequences of sea buckthorn *MADS* analyzed for gene structure were obtained from CNGBdb (https://db.cngb.org/) (Wu et al., 2022). The MADS-box gene structure of sea buckthorn was observed with the TBtools software (Chen et al., 2023), and the count of exons was determined on the exon-intron structural map, which can be found in **Supplementary Table 1**. The protein sequences were analyzed for conserved motifs using MEME (http://meme-suite.org/) (Bailey et al., 2015) with 20 motifs selected. Default parameters were used for the remaining settings, and the diagrams were visualized with TBtools (Chen et al., 2023). Functional networks of MADS-box proteins were examined using STRING (version 12.0) for interacting analysis (Szklarczyk et al., 2023).

### 2.4 Chromosomal localization and synteny analysis

The physical location of the *HrMADS* genes on the chromosome and the chromosome length were determined using TBtools (Chen et al., 2023) based on the location of the gene in the sea buckthorn genome. Tandem duplications are duplications within regions of genes on the same chromosome, whereas segmental duplications are duplications of entire blocks of genes across different chromosomes (Leister, 2014). The *HrMADS* gene was scanned for covariance using the multiple collinear scan toolkit X (MCScanX) in TBtools with default parameters, circos maps were drawn by Advanced circos in TBtools (Chen et al., 2023), and gene duplication events were analyzed based on the covariance data. Use the Simple Ka/Ks Calculator (NG) plugin in TBtools to calculate the ratio of non-synonymous substitution rates (Ka) and synonymous substitution rates (Ks) between two protein-coding genes. This plugin can be used as an indicator of nucleic acid molecular evolution to help determine whether protein-coding genes are affected by selection pressure. In the analysis of syntenic relationships, we retrieved genomic information from four key species [*Arabidopsis* and rice from the EnsemblPlants database (http://plants.ensembl.org/index.html), jujube (*Ziziphus jujuba* Mill.) from the NCBI database (https://www.ncbi.nlm.nih.gov/genome/15586), large-fruited jujube (*Elaeagnus moorcroftⅡ Wall*) from (https://ngdc.cncb.ac.cn/search/?dbId=gwh&q=GWHBJEG00000000&page=1) (Fu et al., 2022)]. The data was subsequently used to generate visualization graphs using TBtools equipped with the Dual Synteny Plotter feature (Chen et al., 2023).

### 2.5 Prediction of cis-acting elements in promoter sequences

An analysis was conducted on the 2000 bp sequence located upstream of the initiation codon of each *HrMADS* gene in order to examine the promoter region and predict cis-acting elements. The promoter sequences were retrieved from CNGBdb (https://db.cngb.org/search/project/CNP0001846/). Subsequently, these promoter sequences were submitted to PlantCARE for the identification of cis-acting elements (http://bioinformatics.psb.ugent.be/webtools/plantcare/html/) as described by Lescot et al. (2002). Utilizing the TBtools software (Chen et al., 2023), graphs were created to illustrate the distribution and functions of the different cis-elements, while Adobe Illustrator tools (McLean, 2002) were employed to enhance the visual presentation of the data.

### 2.6 RNA-seq analysis of *HrMADS* gene expression profile

Based on the climatic observation of sea buckthorn flowering, we divided the flowers of sea buckthorn from dormant buds to fully bloomed flowers into four periods, F1-F4, in which F1 and F2 are the periods of flower buds, F3 is the unfertilized or unpollinated flowers, and F4 is the fully bloomed flowers. To study the expression profile of the *HrMADS* gene associated with the development of male and female flowers in sea buckthorn, we collected F2 and F3 period samples from the sea buckthorn natural hybrid zone located at the eastern edge of the Tibetan Plateau in Qilian County, Qinghai Province, China (38°15′ N latitude, 100°16′ E longitude), which were immediately frozen in liquid nitrogen and then stored at -80°C. Afterwards we sent sea buckthorn male and female flower tissue samples from the F2 and F3 periods to Novogene in Beijing, China, and sequenced them on the Illumina Hiseq platform. The transcript abundance of each gene was calculated from FPKM values (fragments per kilobase repeat per million fragments) (Trapnell et al., 2010). Genes were categorized as follows: those with FPKM values < 1 were considered not expressed, those with FPKM values > 1 were deemed to have low expression, while genes with values exceeding 10 were classified as highly expressed. Genes with FPKM values surpassing 100 were categorized as very highly expressed (Wang et al., 2022). Heat maps of RNA-seq data were visualized using the HeatMap Illustrator tool in TBtools software (Chen et al., 2023).

### 2.7 Expression of some *HrMADS* genes in male, female flowers and floral organs of sea buckthorn by qRT-PCR analysis

Flowering organ samples of sea buckthorn were still collected from the natural hybrid zone of sea buckthorn at the eastern edge of the Tibetan Plateau in Qilian County, Qinghai Province, China (38°15′ N, 100°16′ E), and were well grown. When flowers began to develop and grow, male and female floral organs were sampled separately at the F4 period, with each sample containing three biological replicates. The bracts, pistils, sepals, and stamens of female and male flowers, respectively, were immediately separated and frozen in liquid nitrogen and then stored at -80°C. Total RNA was extracted from male and female flowers and floral organ tissue samples of sea buckthorn using the TIANGEN Polysaccharide Polyphenol Total RNA Extraction Kit (DP441, TIANGEN, China), and first-strand cDNA was synthesized using the Takara PrimeScript RT Reagent Kit with gDNA Eraser (Code No. RR047A, Takara, Dalian). Quantitative RT-PCR assays were performed using QuantStudio 1 Plus (Thermo Fisher Scientific, USA). The 25-μL reaction system consisted of 12.5 μL of TB Green Ex Taq II (No. RR820, Takara), 0.5 μL of ROX Reference Dye II, 1 μL of cDNA, 1 μL of forward primer, 1 μL of reverse primer, and 10 μL of RNase-free water. The qRT-PCR conditions were as follows: 95°C for 30s, 46 cycles of 95°C for 5s and 60°C for 45s, 95°C for 1 min, 60°C for 30 s, 95°C for 30s. The relative gene expression of the *HrMADS* gene was calculated using the 2^-ΔΔCT^ method by normalizing the sea buckthorn gene expression levels based on the endogenous gene 18S (Livak and Schmittgen, 2001). All qRT-PCRs were performed in three biological replicates. Primers are listed in **Supplementary Table 9**.

## 3 Results

### 3.1 Identification and characterization of MADS-box TFs in sea buckthorn

In order to identify the MADS-box gene family, the hidden Markov model (HMM) profile (PF00319) was used along with 107 *Arabidopsis* MADS-box protein sequences, 76 Rice MADS-box protein sequences, and 131 tomato MADS-box protein sequences as queries for HMMER and BLAST searches against the sea buckthorn genome. The MADS-box genes retrieved were subsequently subjected to analysis using NCBI and SMART websites to verify the presence of the complete MADS-box domain. Subsequently, a total of 92 intact MADS-box genes were identified and assigned the names *HrMADS1* to *HrMADS92* based on their chromosomal locations (**Supplementary Table 1**). Subsequently, the physicochemical properties of the *HrMADS* proteins were analyzed in terms of amino acid number (AA), molecular weight (MW), theoretical isoelectric point (pI), instability index (II), and predicted subcellular localization (SL) (**Supplementary Table 1**). The length and molecular weight of the 92 MADS-box proteins ranged from 66 AA and 7721.19 Da (*HrMADS59*) to 475 AA and 53146.47 Da (*HrMADS64*), with isoelectric points ranging from 4.90 (*HrMADS6*) to 10.69 (*HrMADS41*). In addition, 19 MADS proteins were acidic with pI values less than 6.5; 67 were basic with pI values greater than 7.5; and 6 were neutral with pI values between 6.5 and 7.5. The instability index analysis showed that the instability index was 28.55 (*HrMADS17*) - 80.81 (*HrMADS87*), and except for 15 proteins that were stable, the remaining 77 proteins were unstable, with instability indexes greater than 40. These results indicate that the length and molecular weight of *HrMADS* proteins are different and that most of the *HrMADS* proteins are alkaline-unstable proteins. The predicted subcellular localization of the *HrMADS* gene showed that the *HrMADS* gene was distributed in the nucleus (nucl), cytoplasmic matrix (cyto), chloroplast (chlo), cytoplasm and nucleus (cyto-nucl), with most of the *HrMADS* proteins being located in the nucleus (66.30%), and the rest in cytoplasmic matrix (20.66%) and chloroplast (13.04%), which is consistent with the regulatory role of transcription factors in the nucleus. The CDS sequences of *HrMADS* genes ranged from 201 bp (*HrMADS59*) to 1428 bp (*HrMADS64*) (**Supplementary Table 1**). The above results indicated that the physicochemical properties of the sea buckthorn MADS-box family members differed. Secondary structure prediction of *HrMADS* proteins showed that 92 *HrMADS* proteins have alpha helix, extended strand, beta-turn and random coil. Among these structures, alpha helix > random coil > extended strand > beta-turn was predicted in 75 *HrMADS* proteins, and random coil > alpha helix > extended strand > beta-turn was predicted in 17 *HrMADS* proteins (**Supplementary Table 2**).

### 3.2 Phylogenetic analysis and classification of sea buckthorn *HrMADS* genes

**Figure 1:**
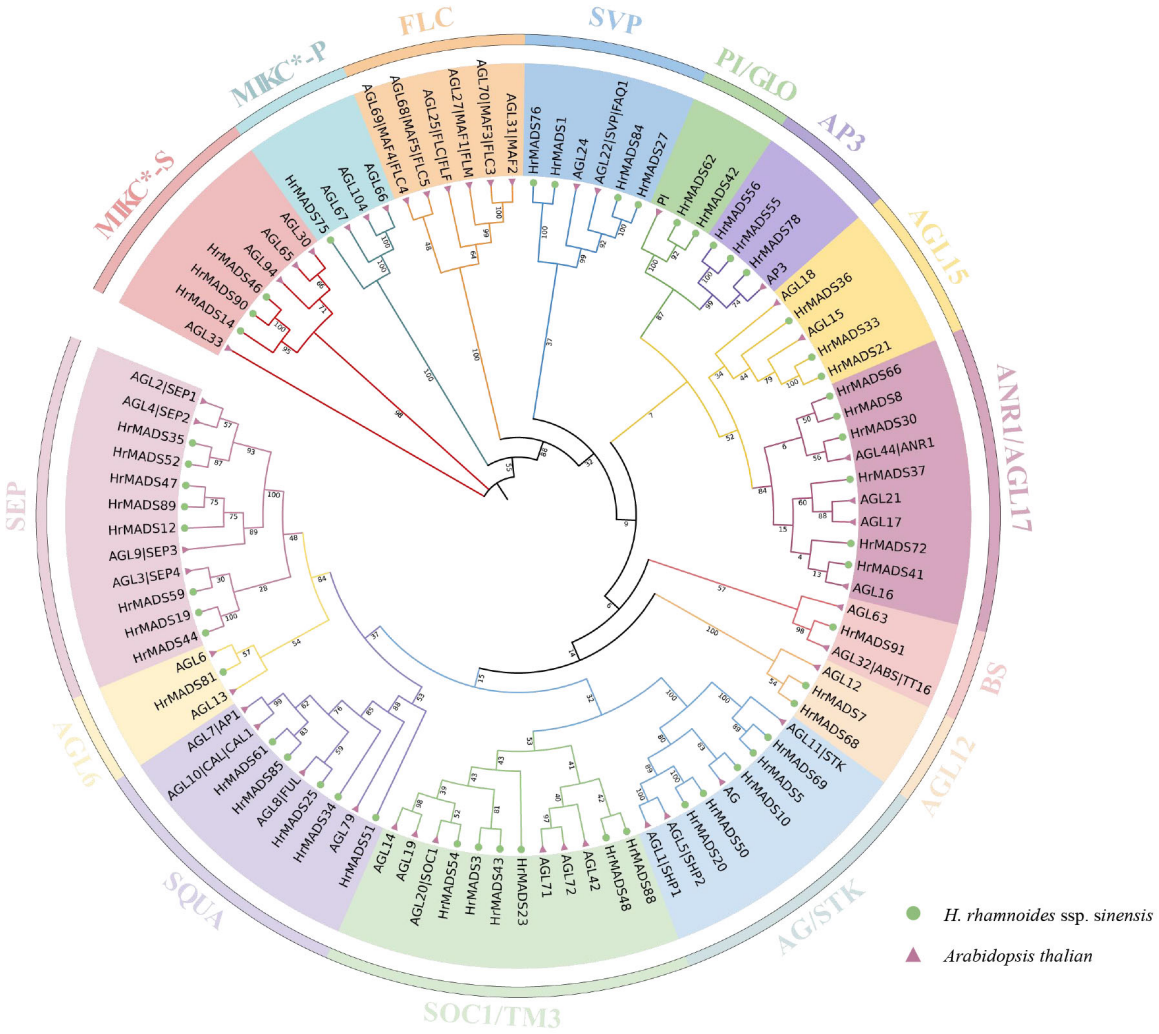
Phylogenetic tree of type II MADS-box genes in *Arabidopsis thaliana* and *H. rhamnoides* ssp. *sinensis*. The phylogenetic trees were constructed using the NJ method. Triangles and circles represent MADS-box proteins in *Arabidopsis thaliana* and sea buckthorn, respectively. Different subfamilies were marked with specific colors and the numbers above the tree are bootstrap values.

To classify sea buckthorn MADS-box proteins and study their evolutionary relationships, we constructed a neighbor-joining (NJ) phylogenetic tree using the results of sequence alignment of sea buckthorn, *Arabidopsis* and rice MADS-box proteins. The results showed that, as in *Arabidopsis*, *HrMADS* genes was categorized into five groups (**Supplementary Figure 1**), of which, 42 sea buckthorn MADS-box genes were categorized as Type I, including 6 Mα genes, 30 Mβ genes and 6 Mγ genes. 50 *HrMADS* genes were categorized as Type II, including 4 MIKC*-type and 46 MIKC^C^-type genes. To further classify the MIKC genes, a neighbor-joining phylogenetic tree (NJ) was constructed using sea buckthorn and *Arabidopsis* MIKC protein sequences (**Figure 1**). Using *Arabidopsis* genes as a reference, the results showed that the sea buckthorn MIKC*-type could be further divided into two subfamilies (MIKC*-s, MIKC*-p), and the MIKC^C^-type was divided into 12 subfamilies [*FLC, SVP, PI(GLO)/AP3, AGL15, AGL17(ANR1), BS, AGL12, AG(STK), SOC1(TM3), SQUA, AGL6*, and *SEP*], and no new subfamilies were identified, indicating evolutionary conservation of genes in dicotyledons. The statistics showed that *HrMADS* genes was distributed among subfamilies, with the *SEP* subfamily containing the most MIKC-type *HrMADS* genes (8), followed by *AGL17* and *SOC1* (*TM3*) with 6 genes each, the *AGL6* and BS subgroups with only one *HrMADS* gene. Significantly, no sea buckthorn genes in the *FLC* subfamily.

### 3.3 Gene structure and conserved motif of sea buckthorn *HrMADS* genes

To assess the diversity of *HrMADS* gene structures, we used TBtools (Chen et al., 2023) to display intron-exon organization. We found that the number of exons ranged from 1 (*HrMADS1*/*7*/*15*/*16*/*26*/*29*/*38*/*58*/*73*/*74*/*76*/*83*/*92*) to 11 (*HrMADS46* and *90*) (**Supplementary Figure 2**, **Supplementary Table 1**). Compared with Type II members, Type I members have fewer introns, especially in the Mα and Mγ subfamilies, and the four MIKC* subgroups *HrMADS* genes (*HrMADS14*, *HrMADS46*, *HrMADS75* and *HrMADS90*) had the highest number of exons. On the other hand, most of the Type II genes have more than five introns, and genes in the same subfamily have similar number of introns, only with different lengths. This indicates that the sea buckthorn MADS-box family’s subfamilies exhibit significant functional similarity, although they may possess distinct regulatory roles.

To characterize the protein structures of the *HrMADS* family, we analyzed the conserved motifs of sea buckthorn *HrMADS* proteins using the MEME program and further annotated these motifs using the SMART analysis website. A total of 20 conserved motifs, named motifs 1–20, were identified among the 92 *HrMADS* protein sequences (**Supplementary Figure 3**, **Supplementary Table 3**). MADS domain and k-domain are key determinants of DNA binding and protein dimerization (Alvarez-Buylla et al., 2000). Motif 1 and motif 3 present typical M domain, and all MIKC^C^ genes contain M domains, whereas the M domain of the majority of the Type I genes are incomplete. The k domain was also detected in a number of Type I proteins, including *HrMADS29* of Mα, *HrMADS 2*, *6*, *13*, *22*, *31*, *44*, *49*, *53*, *60*, *67*, *86*, and *87* of Mβ, this may be an evolutionary specificity of the MADS protein. Motif 4 was only observed in the Mβ subfamily, while motif 7 was only observed in the Mγ subfamily, suggesting that they may be unique to the Mβ and Mγ groups, respectively. Motif 12, 15, and 19 occur only in MIKC*, and these motifs may have important roles in MIKC*. Each subfamily has very similar motif patterns, like *HrMADS* 10, 20, and 50 for the *AG* subfamily and *HrMADS* 25, 34, 51, 61, and 85 for the *SQUA* subfamily, and these proteins may have similar functions. In addition to the M domain (motifs 1 and 3) and K domain (motifs 2, 10, and 13) in the *HrMADS* proteins, MEME searches identified a number of unknown motifs, numbered motifs 4–9, 12, and 14–20. Motif 8 corresponds to the spacer region between the M and K domain, while the remaining motifs are distributed in the C-terminal region. Furthermore, the majority of individuals exhibited robust preservation in the N-terminal region and notable variations in the C-terminal region, mirroring findings observed in MADS-box family proteins within alternative botanical species. This implies a significant level of conservation within this particular gene family across various plant species.

### 3.4 Chromosomal location and gene duplication events of *HrMADS* genes

The chromosomal physical locations of the *HrMADS* genes were determined using the sea buckthorn genome database. 91 *HrMADS* genes were unevenly distributed across the 12 chromosomes of sea buckthorn, with only 1 gene assigned to a non-anchored scaffold (**Supplementary Figure 4**). There are at least four *HrMADS* genes on each chromosome, with the largest number of family members on chromosome 1 with 14 (15.22%), while there are only four genes on chromosome 6. It can be seen that most of the *HrMADS* genes are located at both ends of the chromosome, these results suggest that the distribution of the *HrMADS* family is stochastic and heterogeneous. Tandem duplication events are chromosomal regions containing two or more genes within 200 kb (Holub, 2001). Gene duplication events in the MADS-box family revealed tandem duplication events in four pairs of genes (8.70%), with *HrMADS15*, *HrMADS16* and *HrMADS17*, *HrMADS18* on Chr2, *HrMADS34*, *HrMADS35* on Chr4, and *HrMADS55*, *HrMADS56* on Chr8 (**Supplementary Figure 4**), tandem repetitive events may lead to expansion of family members.

### 3.5 Syntenic analysis of *HrMADS* genes

**Figure 2:**
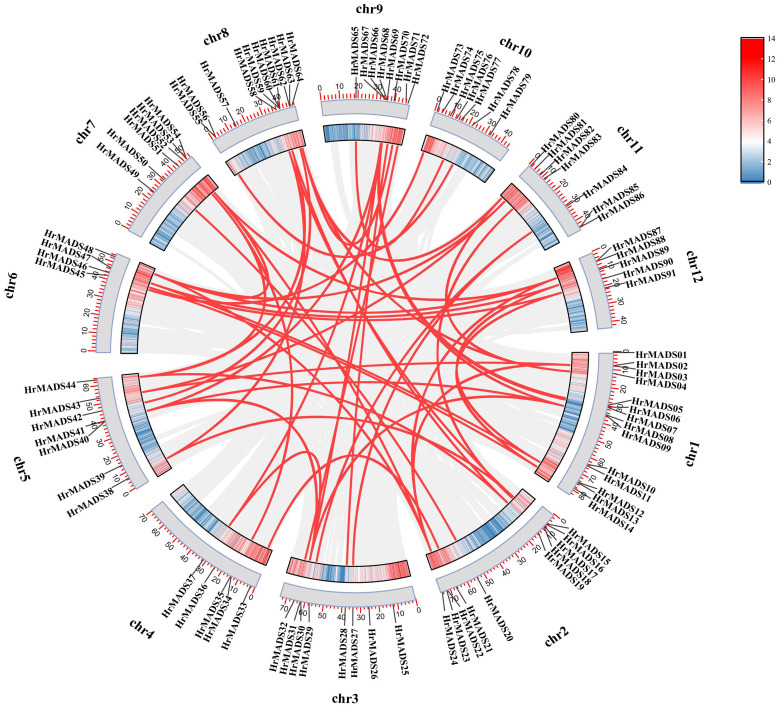
Schematic representation of the interchromosomal relationships and segmental duplication events of the HrMADS genes. Gray lines represent all co-localized blocks in the *H. rhamnoides* ssp. *sinensis* genome, and red lines indicate duplicated MADS gene pairs.

Gene duplication is acknowledged as a catalyst for species evolution, and whole genome duplication (WGD) events may occur in numerous eukaryotes, occasionally multiple times (Van de Peer, 2004). There are three primary categories of gene duplication events: whole genome duplication (WGD), segmental duplication, and tandem duplication (Xu et al., 2012). Among the *HrMADS* genes, in addition to the four tandem duplication events mentioned above, 59 genes (64.13%) were found to be derived from WGD or segmental duplications, 4 genes (4.34%) from tandem duplications, 1 proximal duplicated gene (1.08%), and 28 dispersed duplicated genes (30.43%) (**Figure 2**; **Supplementary Table 1**, **Supplementary Table 4**). In addition, synonymous substitution rates (Ka/Ks) were calculated for gene pairs to assess evolutionary power. Among the 56 pairs of immediate homologous genes, all Ka/Ks values were less than 1 (**Supplementary Table 5**), suggesting that purifying selection may be the main driver of *HrMADS* gene evolution.

**Figure 3:**
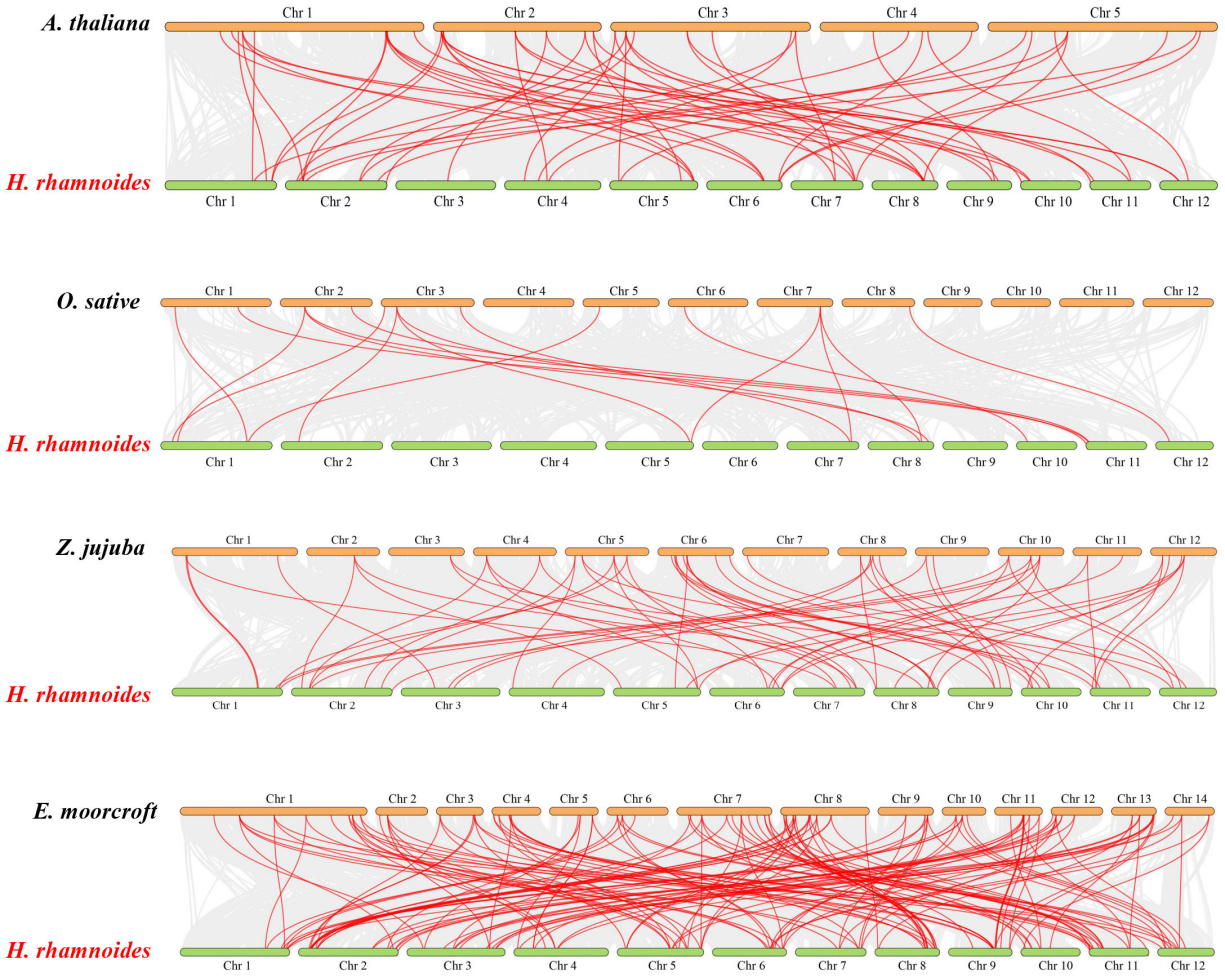
Co-linearity of MADS-box genes in the genomes of *H. rhamnoides* ssp. *sinensis* and *Arabidopsis thalian*, *Oryza sativa* L., *Ziziphus jujuba* Mill. and *Elaeagnus moorcroftII Wall*. Gray lines represent colinear blocks within the two genomes. Red lines highlight co-linear MADS-box gene pairs.

The origin and evolutionary mechanism of the MADS family in sea buckthorn was further deduced by comparing the covariance maps of sea buckthorn with those of four other species (*Arabidopsis*, rice, jujube (*Ziziphus jujuba* Mill.), and large-fruited jujube (*Elaeagnus moorcroftⅡ Wall*)) (**Figure 3**). By genome-wide comparative analysis, 65 *HrMADS* genes were homologous to the MADS genes of the large-fruited jujube, followed by the jujube (46, 50%), *Arabidopsis* (33, 36%), and rice (12, 13%). The number of direct homologous pairs between sea buckthorn and the other four species was 147, 63, 57 and 17 pairs, respectively, and the *HrMADS* gene covariance among dicotyledons was more significant. Many of the co-linear gene pairs were found only in monocotyledonous plants but not in dicotyledonous plants, suggesting that there are evolutionary differences between dicotyledonous plants and monocotyledonous plants. Among them, sea buckthorn and large-fruited jujube had the highest number of co-linear gene pairs, and it is hypothesized that the co-linearity is more significant in these two plants, which belong to the same family, the family Elaeagnaceae, these direct co-lineage gene pairs may have come from the same ancestor.

### 3.6 Prediction and analysis of cis-acting elements in the promoters of *HrMADS* genes

**Figure 4:**
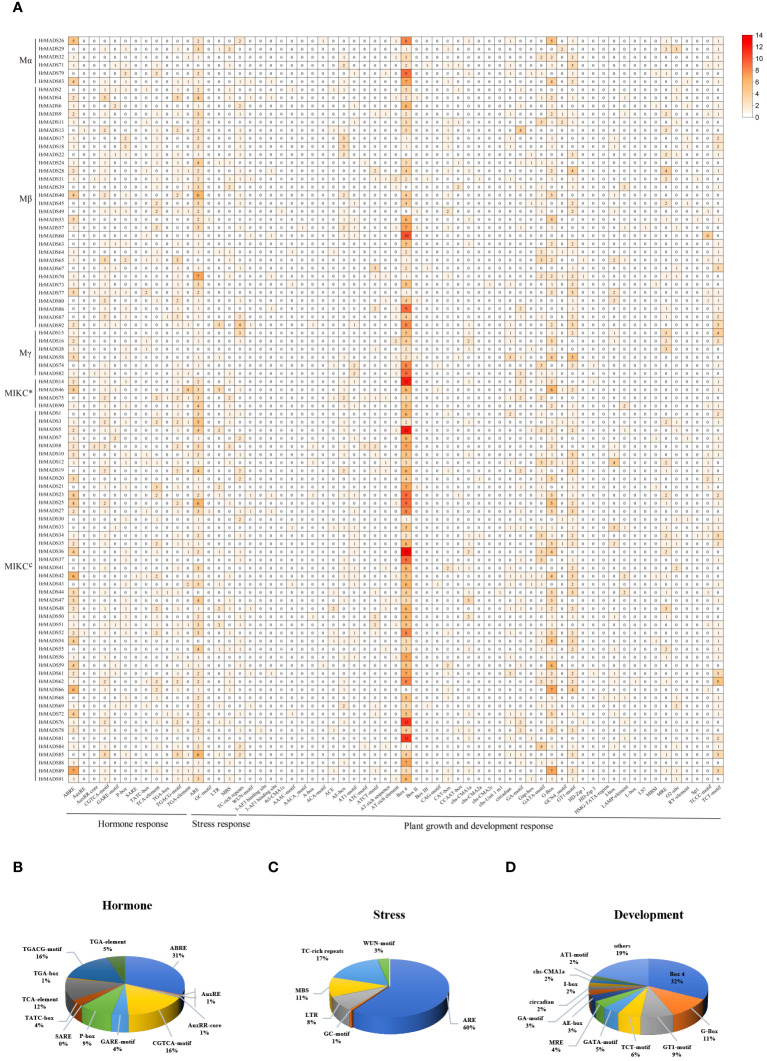
Cis-acting elements of the *HrMADS* gene promoter in *H. rhamnoides* ssp. *sinensis*. **(A)** Number of cis-acting elements of the *HrMADS* gene. **(B)** Total amount of each cis-acting element as a percentage of hormone response elements. **(C)** Total amount of each cis-acting element as a percentage of stress-related elements. **(D)** Total amount of each cis-acting element as a percentage of cis-acting elements involved in plant development and growth.

To explore the regulatory mechanism of *HrMADS* genes, 63 cis-elements were identified in a sequence 2000 bp upstream of the translation start site of each gene, totaling 2,149 cis-acting elements. Three cis-regulatory elements were predicted in each MADS-box promoter, including phytohormones in response to plant growth and development (12, 19.05%), abiotic and biotic stresses (6, 9.52%) and plant growth and development (45,71.43) (**Figure 4**; **Supplementary Tables 6**, **7**).

Among them, 12 hormone response elements were identified in the *HrMADS* gene, including Auxin-related elements (TGA-element, AuxRE, AuxRR-core, and TGA-box), cis-acting elements involved in the response to Sali-cylic Acid (TCA-element and SARE), Abscisic Acid (ABRE), MeJA-related elements (CGTCA-motif and TGACG-motif), and gibberellin-responsive elements (TATC-box, P-box, and GARE-motif). CGTCA-motif, TGACG-motif and ABRE elements were widely distributed in *HrMADS* genes and were found in 89 (96.7%), 89 (96.7%) and 64 (69.5%) *HrMADS* genes, respectively, with the sequences of *HrMADS4*, *HrMADS85*, and *HrMADS89* containing 3 CGTCA-motif, 3 TGACG-motif and 11 ABRE elements (**Figure 4**). These results suggest that the *HrMADS* gene may be associated with MeJA and abscisic acid signaling pathways.

Abiotic stress-related elements included ARE, GC-motif, LTR (low temperature), MBS (drought), TC-rich repeats, and WUN-motif. 31.5% of the *HrMADS* promoter sequences in seabuckthorn contained three or more ARE elements (**Figure 4**). Among them, *HrMADS70*, *HrMADS25*, *HrMADS40*, *HrMADS85*, and *HrMADS3* contained 7, 6, 6, 6, and 5 ARE elements, respectively.

Cis-acting elements involved in plant development and growth are GCN4-motif, AACA_motif and CAT-box, which are involved in endosperm expression and meristematic tissue expression. Also included were light-responsive elements (29,46.03%), circadian regulation of responsiveness (circadian), regulation of protein metabolism (O2-site), and differentiation of the palisade mesophyll cells (HD-Zip 1). About 95.6% of *HrMADS* genes had Box4 light-responsive elements, and half and more of these Box4 elements were distributed in the promoter sequences of *HrMADS37*, *HrMADS60*, *HrMADS81* and *HrMADS88* (**Figure 4**). These results indicate that *HrMADS* genes mainly respond to sea buckthorn in response to environmental changes, hormone response and organ tissue development, and provide a reference for further research on the role of *HrMADS* family genes in the differentiation of sea buckthorn floral organs.

### 3.7 Interaction network of MADS-box TFs between sea buckthorn and *Arabidopsis*

**Figure 5:**
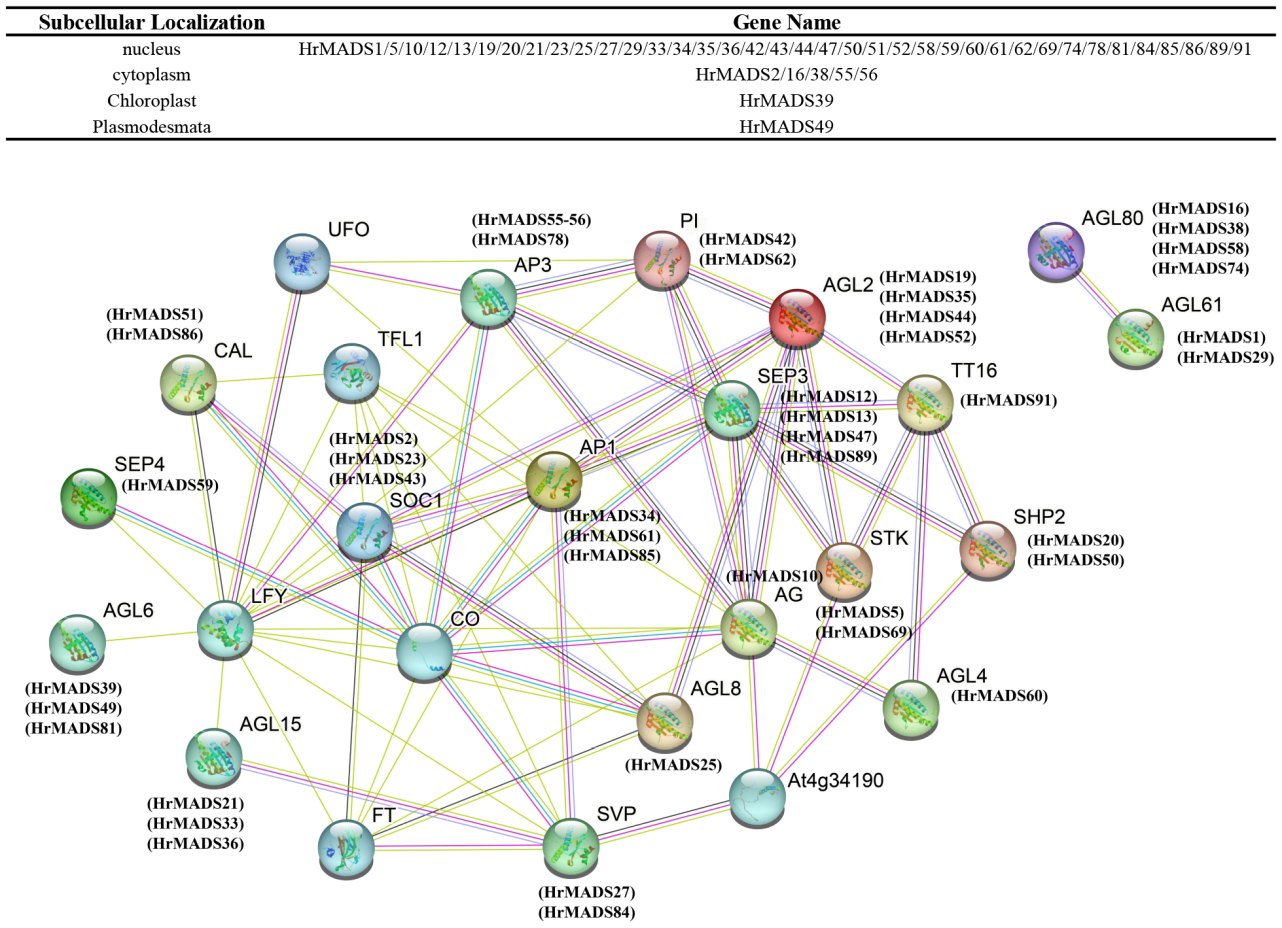
Interaction networks of *HrMADS* in *H. rhamnoides* ssp. *sinensis* based on *Arabidopsis thalian* data and information on the subcellular localization of interacting proteins. Pink line: experimentally determined; green line: gene neighborhood; red line: gene fusions; blue line: gene co-occurrence; cyan line: text mining; black line: co-expression.

Protein-protein interactions play an important role in biological processes in plants, and they provide us with valuable information to help us understand the basis of plant cell function (Gong et al., 2021). The database STRING combines both predicted and existing physical connections and functional relationships among proteins (Szklarczyk et al., 2023). Through the utilization of STRING, we were able to anticipate interactions between sea buckthorn and Arabidopsis proteins in order to form the MADS-box gene network (**Figure 5**). Most of the interacting proteins are located in the nucleus and cytoplasm. Within this network, proteins are symbolized by nodes and variously colored lines illustrate anticipated or established protein interactions. We found that some of the *HrMADS* proteins in our network interacted with flowering-associated proteins, like *AGL8* has the highest protein sequence similarity to *HrMADS25* and interacts with the regulator of flowering time (*CO*), the floral meristem identity determinant protein *LEAFY* (*LFY*), the inflorescence meristem identity determinant protein (*TFL1*), and the phosphatidylethanolamine-binding protein (PEBP) family of proteins (*FT*). Some *HrMADS* genes may be involved in flower development (*AG* and *AP3*), flowering time (*AGL20*), root cell differentiation (*AGL12*), bud development (*AGL15*), embryogenesis (*AGL15*), and early floral meristem identity determination (*LFY*), which plays an important role in floral development and floral organ formation. Whether these genes play a role in the induction and development of male and female flowers in sea buckthorn requires more experiments to confirm. There are also genes (*AGL80* and *AGL61*) that control female gametophyte development, and female plants are less fertile in their absence. These results help us to establish a foundation for future studies that utilize relevant experiments to validate the biological functions of these genes.

### 3.8 Analysis of ABCDE model genes in sea buckthorn

**Figure 6:**
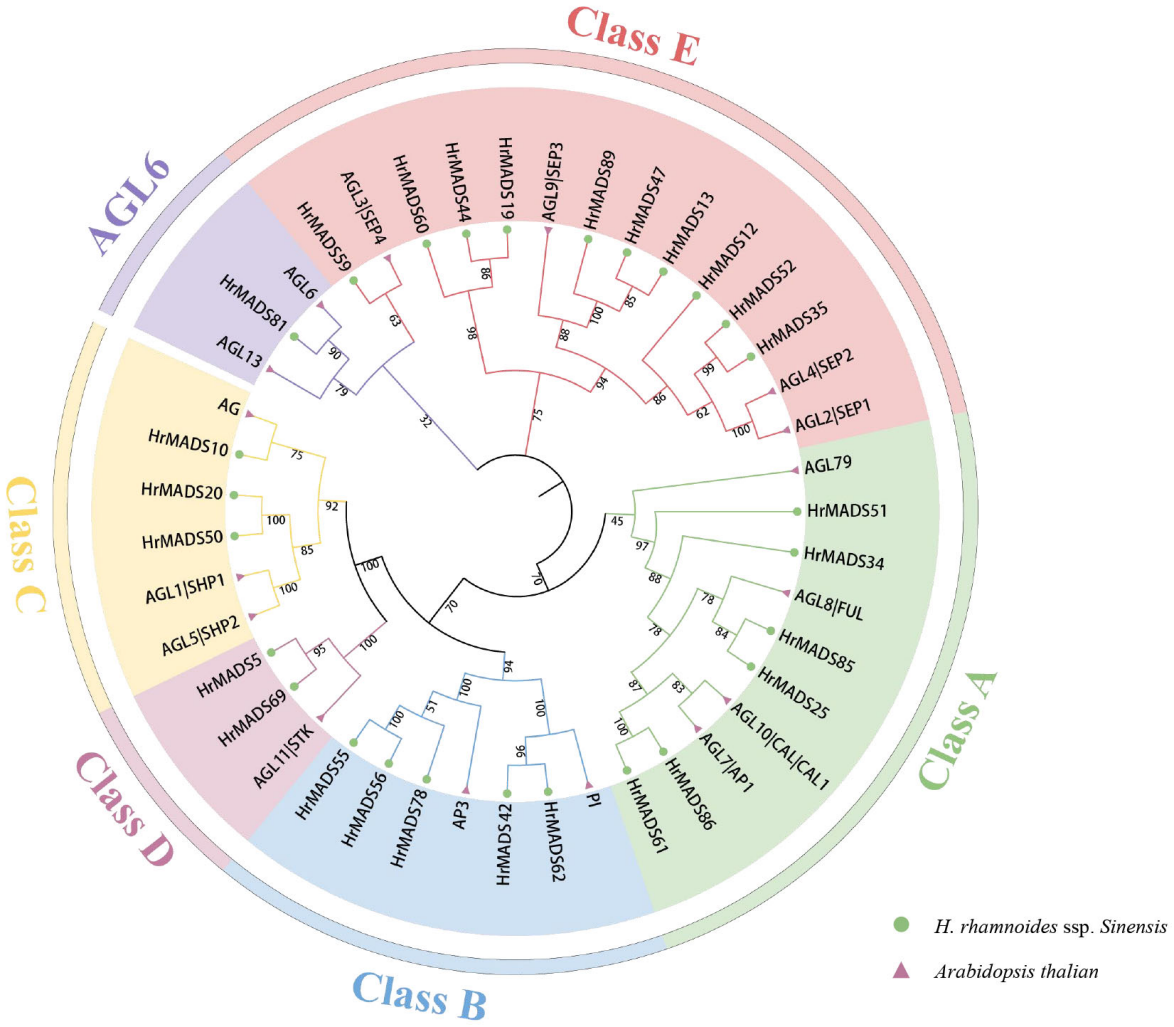
Phylogenetic analysis of ABCDE model genes in *Arabidopsis thalian* and *H. rhamnoides* ssp. *sinensis*. The phylogenetic tree was constructed using the NJ method. Triangles and circles represent MADS-box proteins in *Arabidopsis thaliana* and sea buckthorn. Different subfamilies were marked with specific colors and the numbers above the tree are bootstrap values.

**Figure 7:**
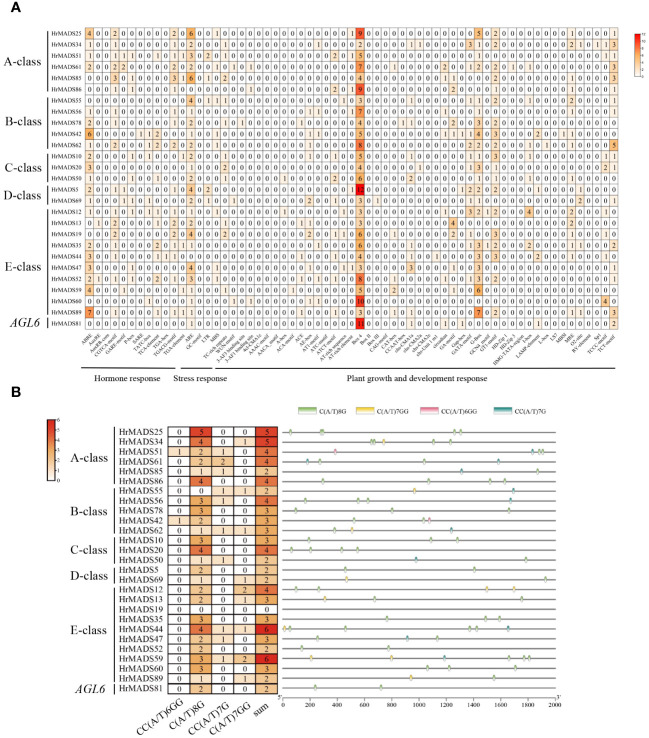
Organization of cis-acting regulatory elements of ABCDE model genes in *H. rhamnoides* ssp. *sinensis*. **(A)** Number of cis-acting elements of ABCDE model genes in *H. rhamnoides* ssp. *sinensis*. **(B)** Number and location of CArG-boxes in each ABCDE model genes.

It has been extensively demonstrated that ABCDE model genes and *AGL6* genes control the development of floral organs. Based on the phylogeny and classification of the model plant *Arabidopsis thaliana*, the ABCDE model genes and *AGL6* genes were characterized in sea buckthorn. As shown in **Figure 6**, we identified a total of 26 ABCDE genes in sea buckthorn, including 6 class A genes (*HrMADS25*/*34*/*51*/*61*/*85*/*86*), 5 class B genes (*HrMADS55*/*56*/*78*/*42*/*62*), 3 class C genes (*HrMADS10*/*20*/*50*), 2 class D genes (*HrMADS5*/*69*), 10 class E genes (*HrMADS13*/*14*/*19*/*35*/*44*/*47*/*52*/*59*/*60*/*89*), and 1 *AGL6* homologous genes (*HrMADS81*). The number and location of cis-acting elements 2000 bp upstream of the promoters of these genes were counted (**Figure 7A**; **Supplementary Table 6**). 20 ABCDE model genes (76.92%) had abscisic acid (ABRE)-responsive elements, and 24 ABCDE model genes (92.31%) had ARE-responsive elements, this suggests that ABCDE model genes may be related to abscisic acid signaling pathway and anaerobic induction. The highest number of cis-acting elements related to light response, such as Box4, G-box, GT1-motif, GATA-motif, and TCT-motif, etc., and all ABCDE model genes possessed Box4 light-responsive elements. 

**Figure 8:**
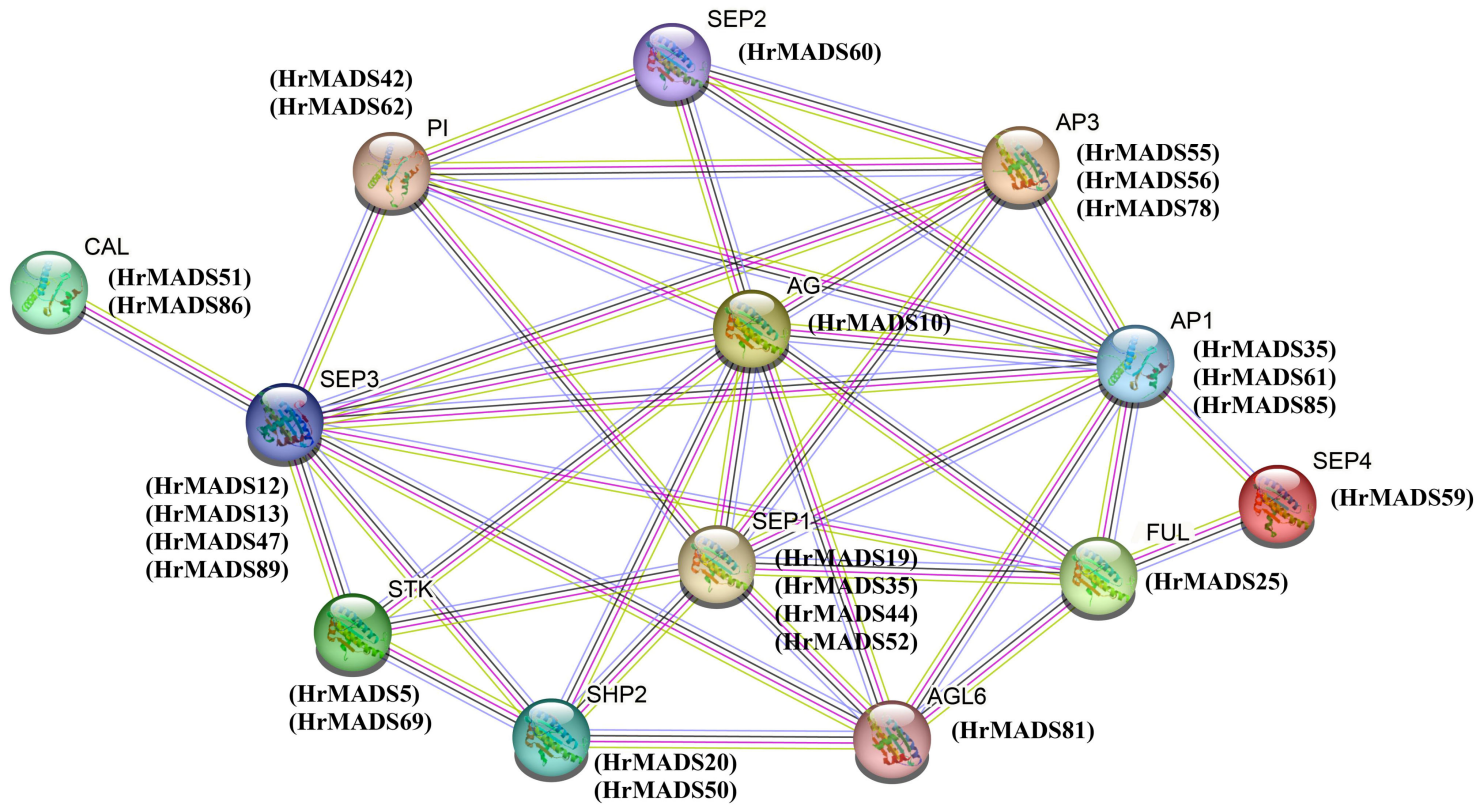
Interaction networks of ABCDE model proteins in *H. rhamnoides* ssp. *sinensis* based on *Arabidopsis thalian* data. Pink line: experimentally determined; green line: gene neighborhood; red line: gene fusions; blue line: gene co-occurrence; cyan line: text mining; black line: co-expression.

A tetramolecular model of floral development states that two MADS proteins form a dimer that binds to a DNA sequence called the “CArG-box” to determine the identity of the floral organ (Gramzow and Theissen, 2010). We counted the number and location of CArG-box on the promoters (**Figure 7B**), with the highest number of CArG-box (6) on *HrMADS44* and *HrMADS59*, and no CArG-box on *HrMADS19*, and finally predicted the interactions between these proteins (**Figure 8**). These interacting proteins are mainly involved in floral meristem development and identity determination, such as *AP1*, *CAL*, *AGL6*, *FUL*, and *SEP4*, as well as in floral organ development and identity determination, such as *PI*, *AP3*, *AG*, *STK*, *SHP1*, *SHP2*, *SEP1*, and *SEP3*. These results will facilitate future studies and validate their biological functions in the development of male and female flowers of sea buckthorn on the basis of relevant experiments.

### 3.9 Expression patterns of the *HrMADS* gene in male and female flowers and floral organs of sea buckthorn

**Figure 9:**
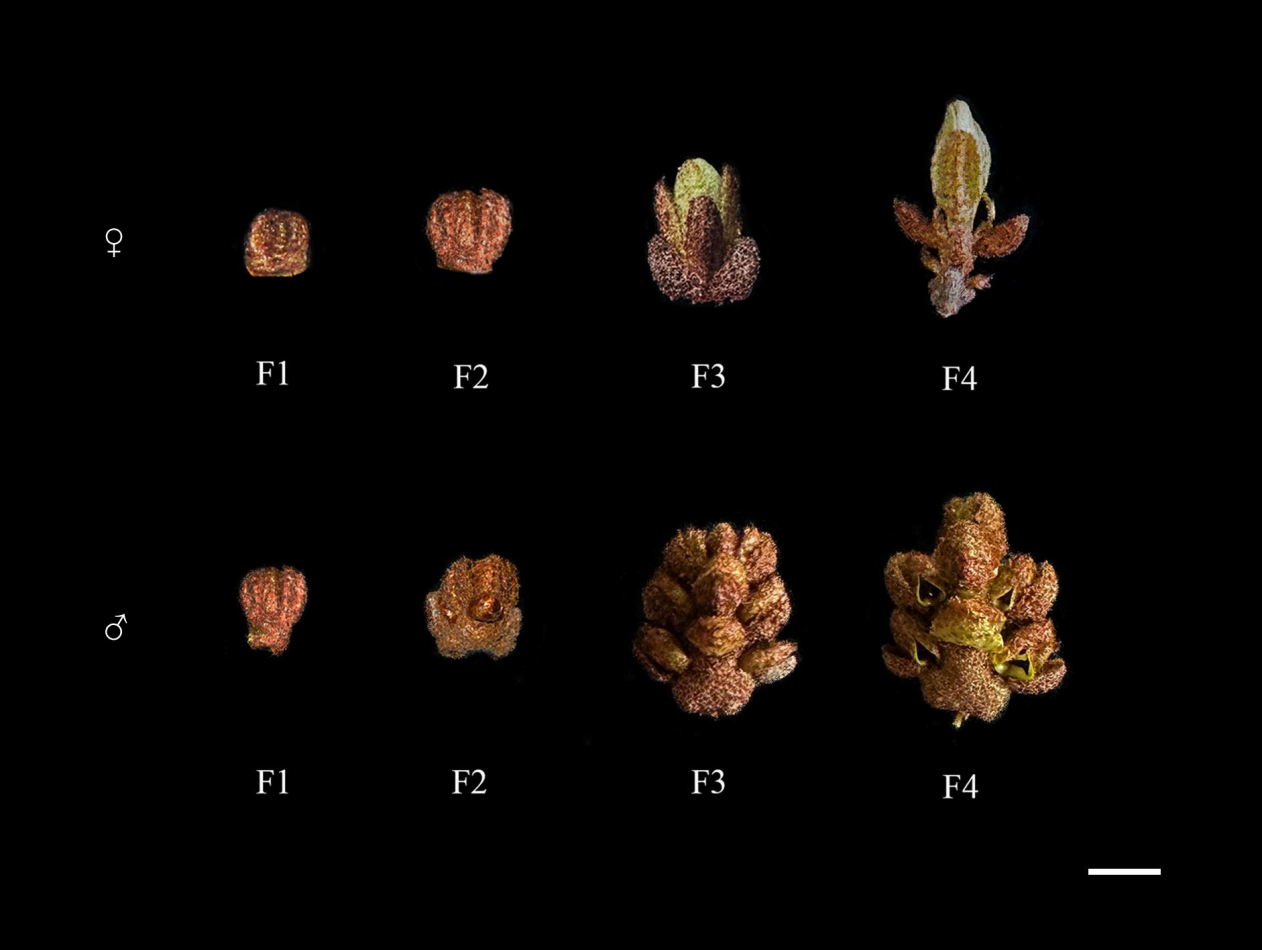
Morphological diagrams of different stages of male and female flower development in H. rhamnoides ssp. sinensis. F1-F4: from the emergence of flower buds to the complete opening of flowers. Bars = 2 mm.

**Figure 10:**
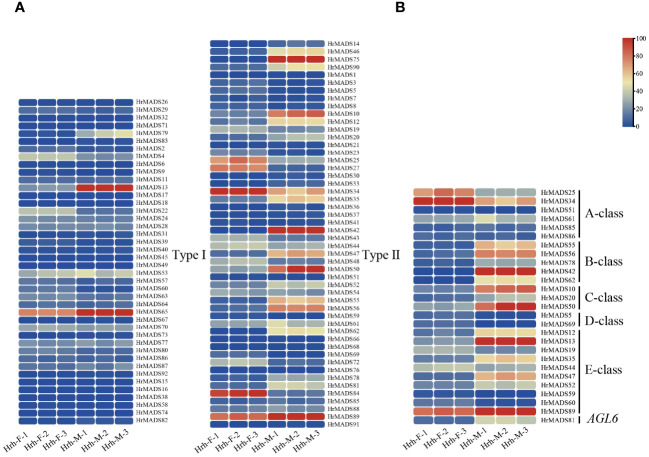
Transcript abundance of the *HrMADS* genes was calculated using FPKM values. **(A)** Expression profiles of *HrMADS* genes in male and female flowers of *H. rhamnoides ssp*. *sinensis*. **(B)** Expression profiles of ABCDE model genes in male and female flowers of *H. rhamnoides ssp*. *sinensis*. Details on expression levels are presented in **Supplementary Tables S7** and **8**.

The MADS-box gene family is widely involved in many key aspects of plant growth and development, and most of the highly expressed MADS genes in flowers are from the Type II subfamily (Ditta et al., 2004). To investigate the expression pattern of the sea buckthorn MADS-box gene, we sent the male and female flower tissue samples of sea buckthorn at F2 and F3 periods to Novogene in Beijing, China, and sequenced them on the Illumina Hiseq platform (**Figure 9**). RNA-seq data of all MADS-box genes as well as ABCDE model genes in male and female flowers of sea buckthorn were analyzed. The results showed that the transcript abundance of *HrMADS* genes varied greatly in male and female flowers of sea buckthorn (**Figure 10A**; **Supplementary Table 7**), and the highly expressed genes were mainly concentrated in the Type II subfamily, which suggests that compared with the Type I *HrMADS* genes, the Type II *HrMADS* genes may play a more important role in flower development. The expression results of ABCDE model genes in male and female flowers of sea buckthorn showed that (**Figure 10B**; **Supplementary Table 8**), as a whole, 27 ABCDE model genes were expressed more in male flowers than in female flowers. Class A genes were expressed in both male and female flowers, class B *HrMADS42* and *HrMADS62* were expressed only in male flowers, and class C genes (*HrMADS10*/*HrMADS20*/*HrMADS50*) were highly expressed in male flowers compared to female flowers, it is hypothesized that class B and C genes are involved in regulating the development of the floral organs of male flowers. Class D genes (*HrMADS5*/*HrMADS69*) are lowly or not expressed in male flowers but highly expressed in female flowers, it is hypothesized that *HrMADS5* and *HrMADS69* are responsible for the confirmation and formation of female floral organs. Almost all E genes were expressed in both male and female flowers, but *HrMADS13* and *HrMADS19* were extremely high in male flowers. These results suggest that ABCDE model genes may be involved in the development of male and female floral organs in sea buckthorn, which is consistent with previous studies on ABCDE model genes (Coen and Meyerowitz, 1991). In addition to the ABCDE genes, *AGL6* has also been shown to control flower development. In wheat, *TaAGL6* has been shown to play an important role in determining stamen development (Murai et al., 2002; Hama et al., 2004; Su et al., 2019). The expression of *HrMADS81*, an *AGL6* homologue in sea buckthorn, was significantly higher in male than in female flowers, suggesting that this gene may also be involved in controlling the development of floral organs in male flowers.

**Figure 11:**
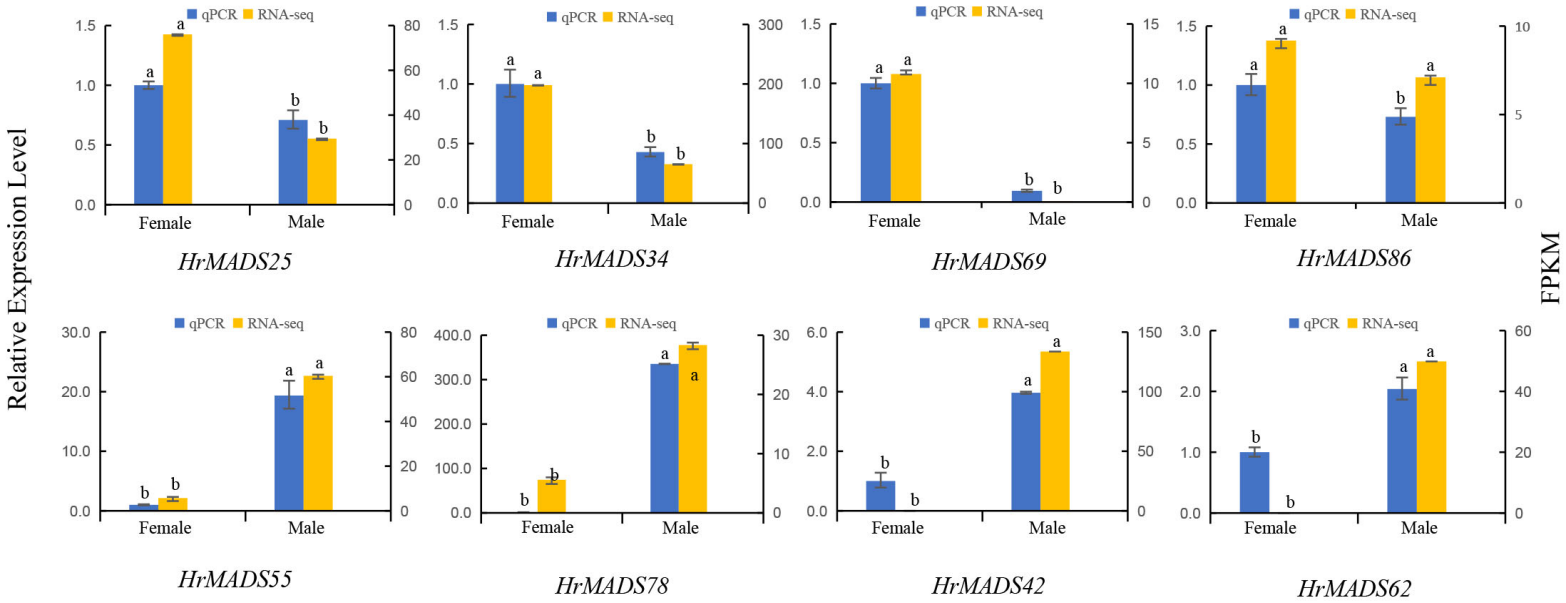
The expression patterns of eight *HrMADS* genes in male and female flowers of *H. rhamnoides ssp. sinensis* were analyzed by qRT-PCR. Error bars represent the standard deviation of three biological replicates, and the data were analyzed by independent samples t-test for significance of differences.

**Figure 12:**
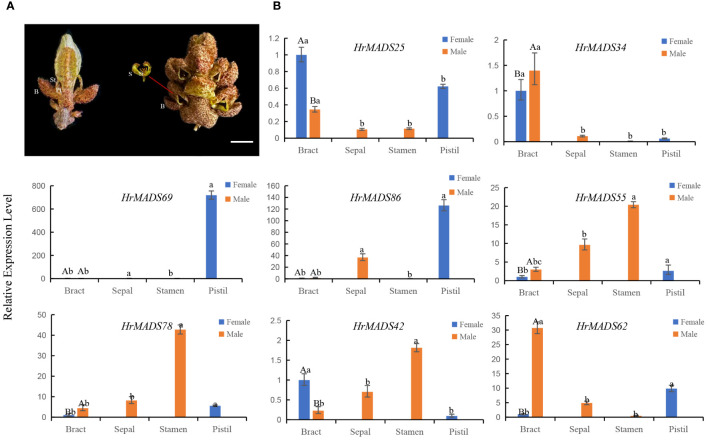
Structure of male and female flowers of sea buckthorn and expression patterns of *HrMADS* genes associated with the development of their floral organs. **(A)** Structure of female and male flowers of sea buckthorn. The female flower consists of a bract (B) and a pistil (St) subtended by a perianth tube (Pt), and the male flower consists of a bract (B), two sepals (S) and four stamens (St). Bars = 2 mm. **(B)** qRT-PCR analysis of the *HrMADS* gene associated with floral organ development in female and male flowers of sea buckthorn. Data were analyzed for significance of differences by one-way ANOVA test (p < 0.05). Bars with the same lowercase letter were not significantly different, while bars with different lowercase letters were significantly different from each other.

Based on the expression profiles of *HrMADS* genes, eight ABCDE model genes highly expressed in flowers were selected for qRT-PCR analysis. The results showed that the expression levels of these genes were basically consistent with the transcriptome sequencing data (**Figure 11**). In addition, transcriptional analysis and qRT-PCR analysis revealed that *HrMADS78* was overexpressed in male flowers, and it was hypothesized that *HrMADS78* plays an important role in the development of male flowers and floral organs. Further validation of the expression of these eight genes in male and female floral organs revealed that the expression patterns of these genes essentially conformed to the traditional ABCDE model classification (**Figure 12**). For example, the expression patterns of four B-type genes (*HrMADS42*/*62*/*55*/*78*) were consistent with the previous ones, in which *HrMADS55*, *78*, and *42* were highly expressed in stamens and *HrMADS62* in male bracts, and these genes may be involved in the determination of the identity of the floral organs in male flowers; the class D gene *HrMADS69* is highly expressed in the pistil and may be involved in pistil development. However, the class A genes *HrMADS25* and *34* were highly expressed in bracts and *HrMADS86* in pistils, which differed from the previous ABCDE model, and these genes may play a role in bract and pistil formation in buckthorn flowers. These results provide a reference for the subsequent study of male and female flower organ determining genes in sea buckthorn as well as the mining of sea buckthorn sex determining genes.

## 4 Discussion

### 4.1 Features of the MADS-box genes in sea buckthorn

MADS-box genes are essential for the formation of flower structures and have received attention in numerous plant species, such as model plant *Arabidopsis thaliana* with 107 genes (Kofuji et al., 2003), rice with 76 genes (Arora et al., 2007); monoecious plant such as camellia with 89 genes (Zhou et al., 2023), lychee with 101 genes (Guan et al., 2021) and *Salvia miltiorrhiza* with 63 genes (Chai et al., 2023); dioecious plants kiwifruit with 89 genes (Ye et al., 2022) and longan with 114 genes (Wang et al., 2022), among others. In this study, we identified 92 *HrMADS* genes in sea buckthorn. The discrepancies in MADS-box gene quantities among species could be linked to genome-wide duplication (WGD) occurrences throughout evolution. Typically, the count of Type II MADS-box genes surpasses that of Type I in the majority of species, as seen in kiwifruit (21 Type I, 68 Type II) (Ye et al., 2022), camellia (38 Type I, 51 Type II) (Zhou et al., 2023), and *Salvia miltiorrhiza* (14 Type I, 49 Type II) (Chai et al., 2023). Sea buckthorn had a larger quantity of Type II *HrMADS* genes (50 genes) compared to Type I (42 genes), possibly because Type II genes underwent a more significant number of duplication occurrences in sea buckthorn. In general, Type II *HrMADS* genes displayed enhanced complexity in gene structures and greater variety in protein structural domains in sea buckthorn. This indicates that Type II *HrMADS* genes might serve a wider range of functions. We categorized the MIKC^C^-type genes in the *HrMADS* genes into 12 subfamilies (**Figure 1**; **Supplementary Table 1**). Sea buckthorn lacking two subfamilies (*FLC* and *OsMADS32*) compared to hexaploid wheat (Schilling et al., 2019), and it lost the *FLC* subfamily compared to *Arabidopsis*. In *Arabidopsis*, *FLC* is a transcription factor that encodes a flowering repressor protein, which directly represses the expression of *FT* (*FLOWERING LOCUS T*) and thus inhibits flowering (Samach et al., 2000). However, in plants like pineapple and rice that do not rely on vernalization for flowering, the FLC branch is notably missing (Arora et al., 2007; Zhang et al., 2020). Sea buckthorn typically flowers in April-May, is not very strict on temperature requirements, and often grows on sunny mountain crests, valleys, dry river, or slopes with gravelly or sandy soils in temperate regions at altitudes of 800–3600 meters. Flowering of sea buckthorn is not dependent on vernalization or cold treatment, and the absence of *FLC* gene in sea buckthorn can also be explained by acclimatization.

### 4.2 Features of the MADS-box genes in sea buckthorn

Analysis of gene structure revealed that most *HrMADS* genes within the same subfamily exhibit similar exon-intron arrangements (**Supplementary Figure 2**). This suggests that the structure and function of *HrMADS* may be conserved during evolution. Within sea buckthorn, motif 1 and 3 contain the characteristic MADS-box transcription factor (*SRF*), which demonstrates a high level of conservation among *HrMADS* genes (**Supplementary Figure 3**). Additionally, the K structural domain is a universally conserved feature in the MADS-box gene family, encompassing motifs 2, 10, and 13. Typically, the K domain is exclusive to the MIKC^C^ subfamily (Parenicová et al., 2003). The k structural domain has also been detected in some non-MIKC^C^ proteins of sea buckthorn, and even in the absence of the k domain, MIKC^C^-type MADS-box proteins still have the ability to bind DNA and function as full-domain proteins (Kaufmann et al., 2005). However, *TaSEP1-A2* proteins are functionally impaired due to deletion of the K domain and are unable to perform protein-protein interactions (Shitsukawa et al., 2007). Further investigation is needed to determine if the diversity of structural domains in MADS-box genes impacts their functionality. Research has shown that the majority of MADS-box genes respond to both phytohormones and various stresses. Numerous cis-acting regulatory elements associated with hormone responses have been discovered (such as ABRE, MeJA, GA, ABRE, etc.) and those related to abiotic stresses (including hypoxic conditions, low temperature, drought, wounding, etc.) in the promoters of *HrMADS* genes. Additionally, a considerable number of cis-acting elements related to plant growth and development were found. Statistically, BOX4 is the most abundant cis-acting element, followed by ARE and ABRE cis-acting elements. ABRE is a cis-acting element associated with the ABA response and plays a role in regulating the expression of ABA-related genes in plants (Yamaguchi-Shinozaki and Shinozaki, 2006). It has been shown that MADS-box genes play important and essential roles in response to different stress conditions (Jian et al., 2017; Dong et al., 2021), which aligns with our findings. The potential biological functions of MADS-box genes warrant further exploration.

### 4.3 Current status of research on sex-determining genes in sea buckthorn

Chawla et al. identified some of the homologous genes involved in *Arabidopsis thaliana* flower development-related genes in sea buckthorn for analyzing potential sex-determining genes in sea buckthorn and analyzed the differential expression of these homologous genes in different flower developmental stages of male and female flowers of sea buckthorn using qRT-PCR. The results indicate that *HrAP1* is specifically expressed in female flowers, whereas *HrAP2* is specifically expressed in male flowers; moreover, therefore, *HrSEP3* and *HrAG* may play a crucial role in determining the floral organ characteristics of male and female flowers, respectively. *HrCRY1*, *HrPHYB* and *HrCO* were highly expressed in male flower development, while *HrCRY2* was highly expressed in all female flower development, and it was hypothesized that the *HrCO* and *HrCRY2* genes might play key roles in male and female flower development and sex determination in sea buckthorn (Chawla et al., 2015). *AG* is a typical class C gene and is essential for the identification of stamens and carpels (Pinyopich et al., 2003). The results of RNA-seq in this study showed that the expression of class C genes *HrMADS10*,*20* and *50* in sea buckthorn was much higher in male flowers than in female flowers, and these genes may play an important role in the development of male and female floral organs of sea buckthorn; the class E genes were expressed to varying degrees in both male and female flowers of sea buckthorn, and they may be involved in the stages of determining the identity of male and female floral organs, which is similar to the results of Chawla et al. In our study, we identified the MADS-box genes of the ABCDE model in sea buckthorn, referred to the traditional ABCDE model to analyze the identity determination of sea buckthorn floral organs and male and female floral sex-determining genes, and did not analyze other genes related to flowering and floral development, such as the homologous genes of *CO*, *FT*, *LFY* and *UFO*, etc. Afterwards, the expression and biological functions of the related genes can be experimentally verified, which can provide a reference for further investigation of the molecular mechanisms of floral organ identity determination and male-female differentiation in male and female flowers of sea buckthorn, but their potential functions still need to be further verified by systematic experiments.

### 4.4 Sea buckthorn ABCDE model genes play a role in the development of male and female floral organs

In lychee, *LcMADS51* (*LcSTK*) plays a positive role in carpel development, whereas six MADS box genes, *LcMADS42*/*46*/*47*/*75*/*93*/*100*, may be involved in stamen development (Guan et al., 2021). In *Salvia miltiorrhiza*, *SmMADS11* is thought to play an important role in regulating anther development and male fertility (Chai et al., 2023). In *Physalis*, *MPF3* specifies calyx identity by interacting with *MPF2* (Zhao et al., 2013), the class B genes *PFGLO1*-*PFDEF* primarily determine corolla and stamen cluster characteristics and play a novel role in gynoecium function (Zhang et al., 2015), and two *PFAG* genes interact to determine male and female organ characteristics and functions (Zhao et al., 2021). MADS-box family genes play a crucial role in the development of male and female floral organs in certain dioecious plant species. In longan, *DlPI* is detected only in petals and stamens, playing a role in petal development and stamen identity determination, and *DlAG* is expressed at a higher level in stamens and carpels than in the other organs, maintaining the function of class C genes in designating male and female reproductive organs (Wang et al., 2022). In kiwifruit, *AcMADS56* and *AcMADS70* might participate in the process of floral differentiation by interacting with the *SyGl* promoter (Ye et al., 2022). In sea buckthorn, a total of 26 ABCDE model-associated *HrMADS* genes were identified, including 6 type A, 5 type B, 3 type C, 2 type D, and 10 type E genes. RNA-seq data showed that class B *HrMADS42* and *HrMADS62* were exclusively expressed in male flowers, which aligns with the expected function of class B genes. Class C genes (*HrMADS10*/*HrMADS20*/*HrMADS50*) exhibited higher expression in male flowers compared to females, suggesting that class B and C genes retain their function in sea buckthorn to regulate stamen development in male flowers. Additionally, class D genes (*HrMADS5*, *HrMADS69*) were highly expressed in female flowers, leading to the hypothesis that *HrMADS5* and *HrMADS69* perform class D functions in female ovule development. Almost all class E genes were expressed in both male and female flowers; however, *HrMADS13* and *HrMADS19* were extremely highly expressed in male flowers, suggesting that they might regulate the development of floral organs at an early stage along with class B and C genes in male flowers.

In rice, the *AGL6* subfamily gene *OsMADS6*, functioning as an E class gene, acts as a cofactor for the formation of higher-order complexes of class A, B, C, and D proteins in all four whorls of the floral organ (Ohmori et al., 2009; Li et al., 2010). In wheat, the role of *TaAGL6* in stamen development has been demonstrated to be significant (Murai et al., 2002; Hama et al., 2004; Su et al., 2019). The expression of *HrMADS81*, the *AGL6* homologous gene in sea buckthorn, was significantly higher in male flowers than in female flowers, suggesting that this gene may also be involved in the controlling stamen development in male flowers. This research also revealed that the MADS-box gene group plays a role in controlling the sexual differences of flowers and identifying the organs in sea buckthorn. Yet, the detailed roles and control mechanisms of ABCDE pattern genes in sea buckthorn require further exploration through experiments.

## 5 Conclusion

In this study, 92 *HrMADS* genes were identified from the sea buckthorn genome. Analysis of their phylogenetic relationships, gene structures, and conserved motifs indicated that most *HrMADS* genes are relatively conserved. Gene duplication is a primary mechanism for gene family expansion and plays a crucial role in biological evolution. The prediction results for cis-acting elements showed that *HrMADS* genes are involved in growth and development, hormone responses (MeJA, ABRE, etc.), and abiotic stresses.

Subsequent analysis of the expression levels of *HrMADS* genes and ABCDE model genes in male and female flowers of sea buckthorn, based on RNA-seq data, revealed distinctive patterns. Notably, the expression level of Type II *HrMADS* genes was significantly higher than that of Type I *HrMADS* genes, suggesting that Type II *HrMADS* genes may play a pivotal role in the development of sea buckthorn flowers. Class B genes (*HrMADS42*, *HrMADS62*) were expressed exclusively in male flower and expression was high in male flower organs. Class C genes (*HrMADS10*, *HrMADS20*, *HrMADS50*) showed higher expression levels in male flowers and The expression was high in male flower organs compared to female flowers, and Class D genes (*HrMADS5*, *HrMADS69*) were low or absent in male flowers and highly expressed in female flowers. The Class E genes (*HrMADS13*, *HrMADS19*) may regulate the development of floral organs in male flowers at an early stage, in conjunction with Class B and C genes. It is hypothesized that these genes are involved in the determination of identity and sexual differentiation of floral organs. The protein interaction network provides clues for identifying related genes.

This study enhances our comprehensive understanding of the characteristics of the MADS-box gene family in sea buckthorn, helps screen *HrMADS* genes involved in the sex differentiation of sea buckthorn, and lays the groundwork for further functional studies and exploration of their mechanisms of action.

## Data availability statement

The data presented in the study are deposited in the GenBank database repository (https://www.ncbi.nlm.nih.gov/), accession number PP400836-PP400927.

## Author contributions

JZ: Conceptualization, Funding acquisition, Methodology, Project administration, Supervision, Writing – review & editing. YX: Data curation, Formal analysis, Investigation, Visualization, Writing – original draft. ZZ: Data curation, Investigation, Writing – original draft. MZ: Data curation, Formal analysis, Writing – review & editing. KL: Investigation, Visualization, Writing – original draft. FW: Data curation, Software, Writing – review & editing. KS: Conceptualization, Funding acquisition, Methodology, Project administration, Supervision, Writing – review & editing.

## References

[B1] Adamczyk B. J. Fernandez D. E. (2009). MIKC* MADS domain heterodimers are required for pollen maturation and tube growth in *Arabidopsis* . Plant Physiol. 149, 1713–1723. doi: 10.1104/pp.109.135806 19211705 PMC2663741

[B2] Agrawal G. K. Abe K. Yamazaki M. Miyao A. Hirochika H. (2005). Conservation of the E-function for floral organ identity in rice revealed by the analysis of tissue culture-induced loss-of-function mutants of the *OsMADS1* gene. Plant Mol. Biol. 59, 125–135. doi: 10.1007/s11103-005-2161-y 16217607

[B3] Alvarez-Buylla E. R. Pelaz S. Liljegren S. J. Gold S. E. Burgeff C. Ditta G. S.. (2000). An ancestral MADS-box gene duplication occurred before the divergence of plants and animals. Proc. Natl. Acad. Sci. U S A. 97, 5328–5333. doi: 10.1073/pnas.97.10.5328 10805792 PMC25828

[B4] Arora R. Agarwal P. Ray S. Singh A. K. Singh V. P. Tyagi A. K.. (2007). MADS-box gene family in rice: genome-wide identification, organization and expression profiling during reproductive development and stress. BMC Genomics 8, 242. doi: 10.1186/1471-2164-8-242 17640358 PMC1947970

[B5] Bailey T. L. Johnson J. Grant C. E. Noble W. S. (2015). The MEME suite. Nucleic Acids Res. 43, W39–W49. doi: 10.1093/nar/gkv416 25953851 PMC4489269

[B6] Becker A. Theissen G. (2003). The major clades of MADS-box genes and their role in the development and evolution of flowering plants. Mol. Phylogenet Evol. 29, 464–489. doi: 10.1016/S1055-7903(03)00207-0 14615187

[B7] Chai S. Li K. Deng X. Wang L. Jiang Y. Liao J.. (2023). Genome-Wide Analysis of the MADS-box Gene Family and Expression Analysis during Anther Development in *Salvia miltiorrhiza* . Int. J. Mol. Sci. 24, 10937. doi: 10.3390/ijms241310937 37446115 PMC10341755

[B8] Chawla A. Stobdan T. Srivastava R. B. Jaiswal V. Chauhan R. S. Kant A. (2015). Sex-Biased Temporal Gene Expression in Male and Female Floral Buds of Sea buckthorn (*Hippophae rhamnoides*). PLoS One 10, e0124890. doi: 10.1371/journal.pone.0124890 25915052 PMC4410991

[B9] Chen C. Wu Y. Li J. Wang X. Zeng Z. Xu J.. (2023). TBtools-II: A "one for all, all for one" bioinformatics platform for biological big-data mining. Mol. Plant 16(11), 1733–1742. doi: 10.1016/j.molp.2023.09.010 37740491

[B10] Coen E. S. Meyerowitz E. M. (1991). The war of the whorls: genetic interactions controlling flower development. Nature. 353, 31–37. doi: 10.1038/353031a0 1715520

[B11] Combet C. Blanchet C. Geourjon C. Deléage G. (2000). NPS@: network protein sequence analysis. Trends Biochem. Sci. 25, 147–150. doi: 10.1016/S0968-0004(99)01540-6 10694887

[B12] Ditta G. Pinyopich A. Robles P. Pelaz S. Yanofsky M. F. (2004). The *SEP4* gene of *Arabidopsis thaliana* functions in floral organ and meristem identity. Curr. Biol. 14, 1935–1940. doi: 10.1016/j.cub.2004.10.028 15530395

[B13] Dong X. Deng H. Ma W. Zhou Q. Liu Z. (2021). Genome-wide identification of the MADS-box transcription factor family in autotetraploid cultivated alfalfa (*Medicago sativa l.*) and expression analysis under abiotic stress. BMC Genomics 22, 603. doi: 10.1186/s12864-021-07911-9 34362293 PMC8348820

[B14] Fernandez-Pozo N. Menda N. Edwards J. D. Saha S. Tecle I. Y. Strickler S. R.. (2015). The Sol Genomics Network (SGN)–from genotype to phenotype to breeding. Nucleic Acids Res. 43, D1036–D1041. doi: 10.1093/nar/gku1195 25428362 PMC4383978

[B15] Folter S. D. Angenent G. C. (2006). Trans meets cis in MADS science. Trends Plant Sci. 11, 224–231. doi: 10.1016/j.tplants.2006.03.008 16616581

[B16] Fu X. Wu J. Ma X. Li K. Zhang H. Wu S.. (2022). The chromosome-level genome of *Elaeagnus moorcroftii Wall.*, an economically and ecologically important tree Species in drylands. Diversity. 14, 468. doi: 10.3390/d14060468

[B17] Garcia-Hernandez M. Berardini T. Z. Chen G. Crist D. Doyle A. Huala E.. (2002). TAIR: a resource for integrated *Arabidopsis* data. Funct. Integr. Genomics 2, 239–253. doi: 10.1007/s10142-002-0077-z 12444417

[B18] Gong F. Cao D. Qu C. Yin D. Zhao Q. Xiong E. (2021). Advances in the elucidation of nuclear proteins in the model plant *Arabidopsis thaliana*: Basedon protein interactions and bioinformatics analysis. J. Plant Interact. 16, 481–493. doi: 10.1080/17429145.2021.1998681

[B19] Gramzow L. Theissen G. (2010). A hitchhiker's guide to the MADS world of plants. Genome Biol. 11, 214. doi: 10.1186/gb-2010-11-6-214 20587009 PMC2911102

[B20] Gramzow L. Theissen G. (2015). Phylogenomics reveals surprising sets of essential and dispensable clades of mikc(c) -group mads-box genes in flowering plants. J. Exp. Zoology Part B: Mol. Dev. Evol. 324, 353–362. doi: 10.1002/jez.b.22598 25678468

[B21] Guan H. Wang H. Huang J. Liu M. Chen T. Shan X.. (2021). Genome-wide identification and expression analysis of MADS-box family genes in litchi (*Litchi chinensis* sonn.) and their involvement in floral sex determination. Plants (Basel). 10, 2142. doi: 10.3390/plants10102142 34685951 PMC8540616

[B22] Hama E. Takumi S. Ogihara Y. Murai K. (2004). Pistillody is caused by alterations to the class-B MADS-box gene expression pattern in alloplasmic wheats. Planta. 218, 712–720. doi: 10.1007/s00425-003-1157-6 14652757

[B23] Holub E. B. (2001). The arms race is ancient history in *Arabidopsis*, the wildflower. Nat. Rev. Genet. 2, 516. doi: 10.1038/35080508 11433358

[B24] Horton P. Park K. J. Obayashi T. Fujita N. Harada H. Adams-Collier C. J.. (2007). WoLF PSORT: protein localization predictor. Nucleic Acids Res. 35, W585–W587. doi: 10.1093/nar/gkm259 17517783 PMC1933216

[B25] Jian M. Yang Y. Luo W. Yang C. Ding P. Liu Y.. (2017). Genome-wide identification and analysis of the MADS-box gene family in bread wheat (*Triticum aestivum* L.). PLoS One 12 (7), e0181443. doi: 10.1371/journal.pone.0181443 28742823 PMC5526560

[B26] Kaufmann K. Melzer R. TheißEn G. (2005). MIKC-type MADS-domain proteins: Structural modularity, protein interactions and network evolution in land plants. Gene. 347, 183–198. doi: 10.1016/j.gene.2004.12.014 15777618

[B27] Kawahara Y. de la Bastide M. Hamilton J. P. Kanamori H. McCombie W. R. Ouyang S.. (2013). Improvement of the *Oryza sativa* Nipponbare reference genome using next generation sequence and optical map data. Rice (N Y). 6 (1), 4. doi: 10.1186/1939-8433-6-4 24280374 PMC5395016

[B28] Kofuji R. Sumikawa N. Yamasaki M. Kondo K. Ueda K. Ito M.. (2003). Evolution and divergence of the MADS-box gene family based on genome-wide expression analyses. Mol. Biol. Evol. 20, 1963–1977. doi: 10.1093/molbev/msg216 12949148

[B29] Leister D. (2014). Tandem and segmental gene duplication and recombination in the evolution of plant disease resistance genes. Trends Genet. 20, 116–122. doi: 10.1016/j.tig.2004.01.007 15049302

[B30] Lescot M. Deíhais P. Thijs G. Marchal K. Moreau Y. Van de Peer Y.. (2002). PlantCARE, a database of plant cis-acting regulatory elements and a portal to tools for in silico analysis ofpromoter sequences. Nucleic Acids Res. 30, 325–327. doi: 10.1093/nar/30.1.325 11752327 PMC99092

[B31] Letunic I. Bork P. (2018). 20 years of the SMART protein domain annotation resource. Nucleic Acids Res. 46(D1), D493–D496. doi: 10.1093/nar/gkx922 29040681 PMC5753352

[B32] Letunic I. Bork P. (2021). Interactive Tree Of Life (iTOL) v5: an online tool for phylogenetic tree display and annotation. Nucleic Acids Res. 49, W293–W296. doi: 10.1093/nar/gkab301 33885785 PMC8265157

[B33] Li H. Liang W. Jia R. Yin C. Zong J. Kong H.. (2010). The *AGL6*-like gene *OsMADS6* regulates floral organ and meristem identities in rice. Cell Res. 20, 299–313. doi: 10.1038/cr.2009.143 20038961

[B34] Livak K. J. Schmittgen T. D. (2001). Analysis of relative gene expression data using real-time quantitative PCR and the 2^-ΔΔCT^ method. Methods 25, 402–408. doi: 10.1006/meth.2001.1262 11846609

[B35] Ma H. (2000). The ABCs offloral evolution. Cell 101, 5–8. doi: 10.1016/S0092-8674(00)80618-2 10778850

[B36] Marchler-Bauer A. Derbyshire M. K. Gonzales N. R. Lu S. Chitsaz F. Geer L. Y.. (2015). CDD: NCBI’s conserved domain database. Nucleic Acids Res. 43, D222–D226. doi: 10.1093/nar/gku1221 25414356 PMC4383992

[B37] Masiero S. Colombo L. Grini P. E. Schnittger A. Kater M. M. (2011). The emerging importance of type I MADS box transcription factors for plant reproduction. Plant Cell. 23, 865–872. doi: 10.1105/tpc.110.081737 21378131 PMC3082269

[B38] McLean D. (2002). Adobe photoshop and illustrator techniques. J. Audiov. Media Med. 25, 79–81. doi: 10.1080/01405110220140865 12150034

[B39] Messenguy F. Dubois E. (2003). Role of MADS box proteins and their cofactors in combinatorial control of gene expression and cell development. Gene. 316, 1–21. doi: 10.1016/S0378-1119(03)00747-9 14563547

[B40] Mistry J. Chuguransky S. Williams L. Qureshi M. Salazar G. A. Sonnhammer E. L. L.. (2021). Pfam: The protein families database in 2021. Nucleic Acids Res. 49, D412–D419. doi: 10.1093/nar/gkaa913 33125078 PMC7779014

[B41] Münster T. Pahnke J. Di Rosa A. Kim J. T. Martin W. Saedler H.. (1997). Floral homeotic genes were recruited from homologous MADS-box genes preexisting in the common ancestor of ferns and seed plants. Proc. Natl. Acad. Sci. U S A. 94, 2415–2420. doi: 10.1073/pnas.94.6.2415 9122209 PMC20102

[B42] Murai K. Takumi S. Koga H. Ogihara Y. (2002). Pistillody, homeotic transformation of stamens into pistil-like structures, caused by nuclear-cytoplasm interaction in wheat. Plant J. 29, 169–181. doi: 10.1046/j.0960-7412.2001.01203.x 11851918

[B43] Ohmori S. Kimizu M. Sugita M. Miyao A. Hirochika H. Uchida E.. (2009). *MOSAIC FLORAL ORGANS1*, an *AGL6-like* MADS box gene, regulates floral organ identity and meristem fate in rice. Plant Cell. 21, 3008–3025. doi: 10.1105/tpc.109.068742 19820190 PMC2782282

[B44] Parenicová L. de Folter S. Kieffer M. Horner D. S. Favalli C. Busscher J.. (2003). Molecular and phylogenetic analyses of the complete MADS-box transcription factor family in *Arabidopsis*: new openings to the MADS world. Plant Cell. 15, 1538–1551. doi: 10.1105/tpc.011544 12837945 PMC165399

[B45] Pinyopich A. Ditta G. S. Savidge B. Liljegren S. J. Baumann E. Wisman E.. (2003). Assessing the redundancy of MADS-box genes during carpel and ovule development. Nature 424, 85–88. doi: 10.1038/nature01741 12840762

[B46] Samach A. Onouchi H. Gold S. E. Ditta G. S. Schwarz-Sommer Z. Yanofsky M. F.. (2000). Distinct roles of CONSTANS target genes in reproductive development of *Arabidopsis* . Science. 288(5471), 1613–1616. doi: 10.1126/science.288.5471.1613 10834834

[B47] Schilling S. Kennedy A. Pan S. Jermiin L. S. Melzer R. (2019). Genome-wide analysis of MIKC-type MADS-box genes in wheat: pervasive duplications, functional conservation and putative neofunctionalization. New Phytol. 225, 511–529. doi: 10.1111/nph.16122 31418861

[B48] Shitsukawa N. Ikari C. Mitsuya T. Sakiyama T. Murai K. (2007). Wheat *SOC1* functions independently of *WAP1*/*VRN1*, an integrator of vernalization and photoperiod flowering promotion pathways. Physiologia Plantarum. 130, 627–636. doi: 10.1111/j.1399-3054.2007.00927.x

[B49] Su Y. Liu J. Liang W. Dou Y. Fu R. Li W.. (2019). Wheat *AGAMOUS LIKE 6* transcription factors function in stamen development by regulating the expression of *TaAPETALA3* . Development 146, dev177527. doi: 10.1242/dev.177527 31540915

[B50] Szklarczyk D. Kirsch R. Koutrouli M. Nastou K. Mehryary F. Hachilif R.. (2023). The STRING database in 2023: protein-protein association networks and functional enrichment analyses for any sequenced genome of interest. Nucleic Acids Res. 51(D1), D638–D646. doi: 10.1093/nar/gkac1000 36370105 PMC9825434

[B51] Tamura K. Stecher G. Kumar S. (2021). MEGA11: molecular evolutionary genetics analysis version 11. Mol. Biol. Evol. 38(7), 3022–3027. doi: 10.1093/molbev/msab120 33892491 PMC8233496

[B52] Theißen G. (2001). Development of floral organ identity: stories from the MADS house. Curr. Opin. Plant Biol. 4, 75–85. doi: 10.1016/S1369-5266(00)00139-4 11163172

[B53] Theissen G. Becker A. Di R. A. Kanno A. Kim J. T. Münster T.. (2000). A short history of MADS-box genes in plants. Plant Mol. Biol. 42, 115–149. doi: 10.1023/A:1006332105728 10688133

[B54] Trapnell C. Williams B. A. Pertea G. Mortazavi A. Kwan G. Baren M. J.. (2010). Transcript assembly and quantification by RNA-seq reveals unannotated transcripts and isoform switching during cell differentiation. Nat. Biotechnol. 28, 511–515. doi: 10.1038/nbt.1621 20436464 PMC3146043

[B55] Van de Peer Y. (2004). Computational approaches to unveiling ancient genome duplications. Nat. Rev. Genet. 5, 752–763. doi: 10.1038/nrg1449 15510166

[B56] Wang B. Hu W. Fang Y. Feng X. Fang J. Zou T.. (2022). Comparative analysis of the MADS-box genes revealed their potential functions for flower and fruit development in longan (*Dimocarpus longan*). Front. Plant Sci. 12, 813798. doi: 10.3389/fpls.2021.813798 35154209 PMC8829350

[B57] Wilkins M. R. Gasteiger E. Bairoch A. Sanchez J. C. Williams K. L. Appel R. D.. (1999). Protein identification and analysis tools in the ExPASy server. Methods Mol. Biol. 112, 531–552. doi: 10.1385/1-59259-584-7:531 10027275

[B58] Wu Z. Chen H. Pan Y. Feng H. Fang D. Yang J.. (2022). Genome of *Hippophae rhamnoides* provides insights into a conserved molecular mechanism in actinorhizal and rhizobial symbioses. New Phytol. 235, 276–291. doi: 10.1111/nph.18017 35118662

[B59] Xu G. Guo C. Shan H. Kong H. (2012). Divergence of duplicate genes in exon-intron structure. Proc. Natl. Acad. Sci. U S A. 109, 1187–1192. doi: 10.1073/pnas.1109047109 22232673 PMC3268293

[B60] Yamaguchi-Shinozaki K. Shinozaki K. (2006). Transcriptional regulatory networks in cellular responses and tolerance to dehydration and cold stresses. Annu. Rev. Plant Biol. 57, 781–803. doi: 10.1146/annurev.arplant.57.032905.105444 16669782

[B61] Ye L. X. Luo M. M. Wang Z. Bai F. X. Luo X. Gao L.. (2022). Genome-wide analysis of MADS-box gene family in kiwifruit (*Actinidia chinensis* var. *chinensis*) and their potential role in floral sex differentiation. Front. Genet. 13. doi: 10.3389/fgene.2022.1043178 PMC9714460 36468015

[B62] Zhang C. (2006). Investigation on flower and fruit drop of superior sea buckthorn trees. Shanxi For Sci. Technol. 04), 42–43. doi: 10.3969/j.issn.1007-726X.2006.04.016

[B63] Zhang X. Fatima M. Zhou P. Ma Q. Ming R. (2020). Analysis of MADS-box genes revealed modified flowering gene network and diurnal expression in pineapple. BMC Genomics 21, 8. doi: 10.1186/s12864-019-6421-7 31896347 PMC6941321

[B64] Zhang S. Zhang J. S. Zhao J. He C. (2015). Distinct subfunctionalization and neofunctionalization of the B-class MADS-box genes in *Physalis floridana* . Planta. 241, 387–402. doi: 10.1007/s00425-014-2190-3 25326772

[B65] Zhao J. Gong P. Liu H. Zhang M. He C. (2021). Multiple and integrated functions of floral C-class MADS-box genes in flower and fruit development of *Physalis floridana* . Plant Mol. Biol. 107, 101–116. doi: 10.1007/s11103-021-01182-4 34424500

[B66] Zhao J. Tian Y. Zhang J. S. Zhao M. Gong P. Riss S.. (2013). The euAP1 protein MPF3 represses *MPF2* to specify floral calyx identity and displays crucial roles in Chinese lantern development in *Physalis* . Plant Cell. 5, 2002–2021. doi: 10.1105/tpc.113.111757 PMC3723609 23792370

[B67] Zhou P. Qu Y. Wang Z. Huang B. Wen Q. Xin Y.. (2023). Gene structural specificity and expression of MADS-box gene family in *camellia chekiangoleosa* . Int. J. Mol. Sci. 24, 3434. doi: 10.3390/ijms24043434 36834845 PMC9960327

