# Supplementary material for: Genome-wide analysis of the MADS-box gene family of sea buckthorn (Hippophae rhamnoides ssp. sinensis) and their potential role in floral organ development
Source: Front Plant Sci. 2024 Jun 13;15:1387613. doi: 10.3389/fpls.2024.1387613 (PMC11208494; doi:10.3389/fpls.2024.1387613)
Supplement: Supplementary file 1 [file Image_1.pdf]

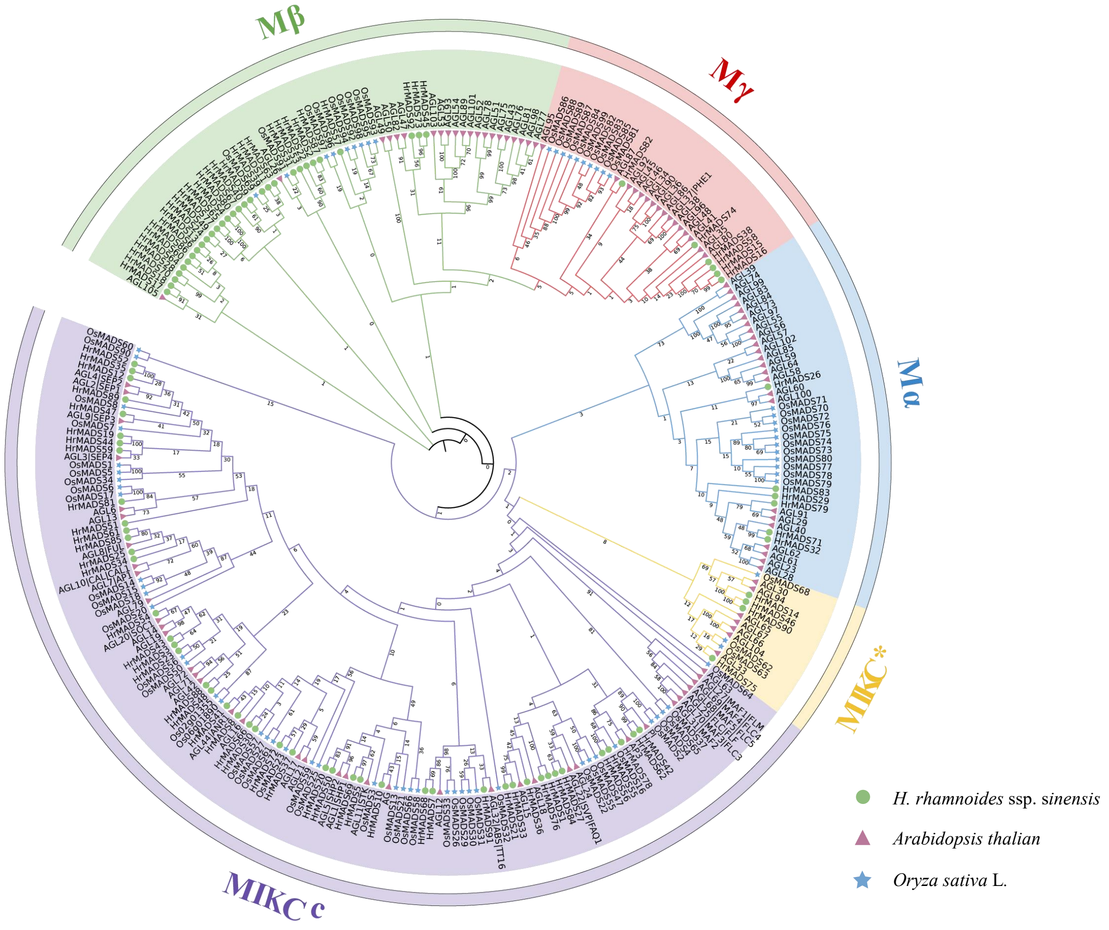

Supplementary Figure 1. Phylogenetic tree of MADS-box genes in *Arabidopsis thalian*, *Oryza sativa* L., and *H. rhamnoides* ssp. s*inensis*. The phylogenetic tree was constructed using the NJ method. Triangles represent MADS-box proteins in Arabidopsis, and blue stars and circles represent MADS-box proteins in rice and sea buckthorn, respectively. Different subfamilies are marked with specific colors.

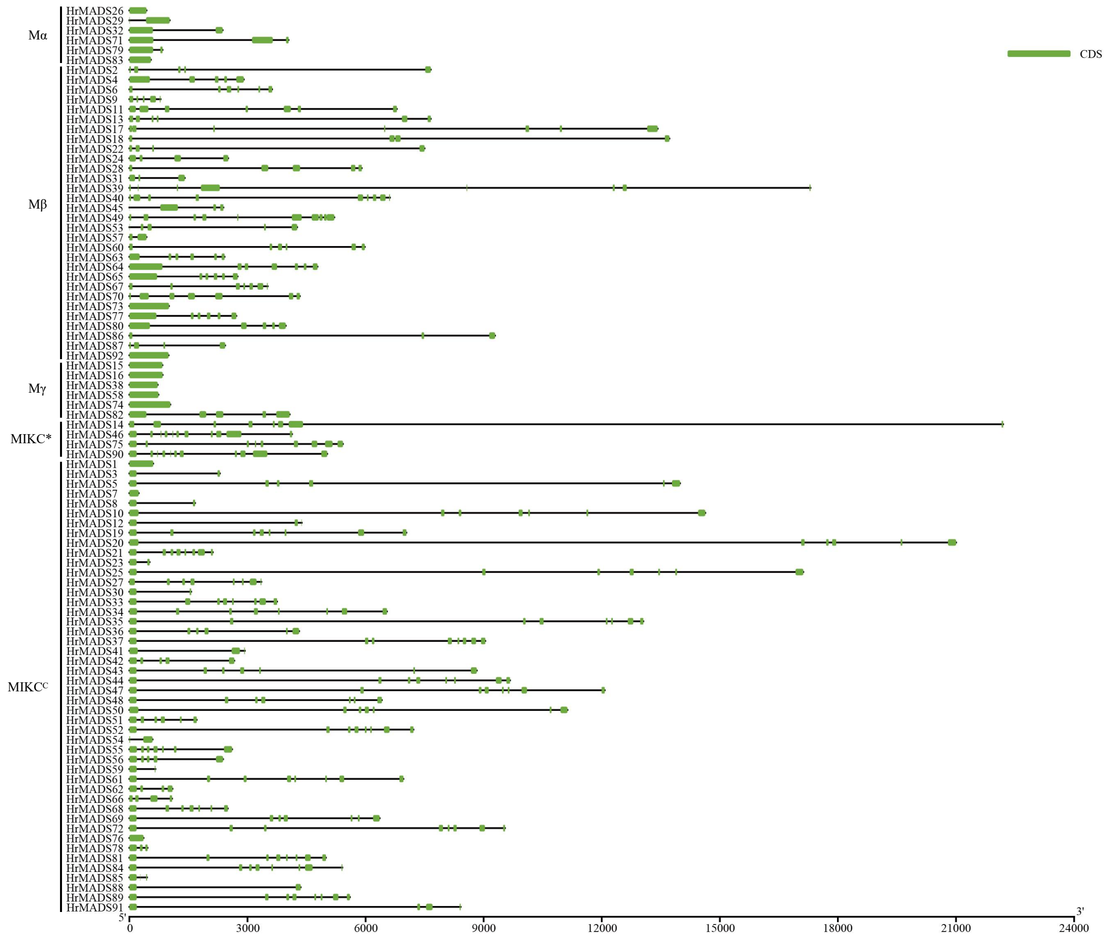

Supplementary Figure 2. Gene structure of the *HrMADS* genes. Green boxes indicate exons and gray lines indicate introns. The detailed number of exons per gene is shown in Supplemental Table S1.

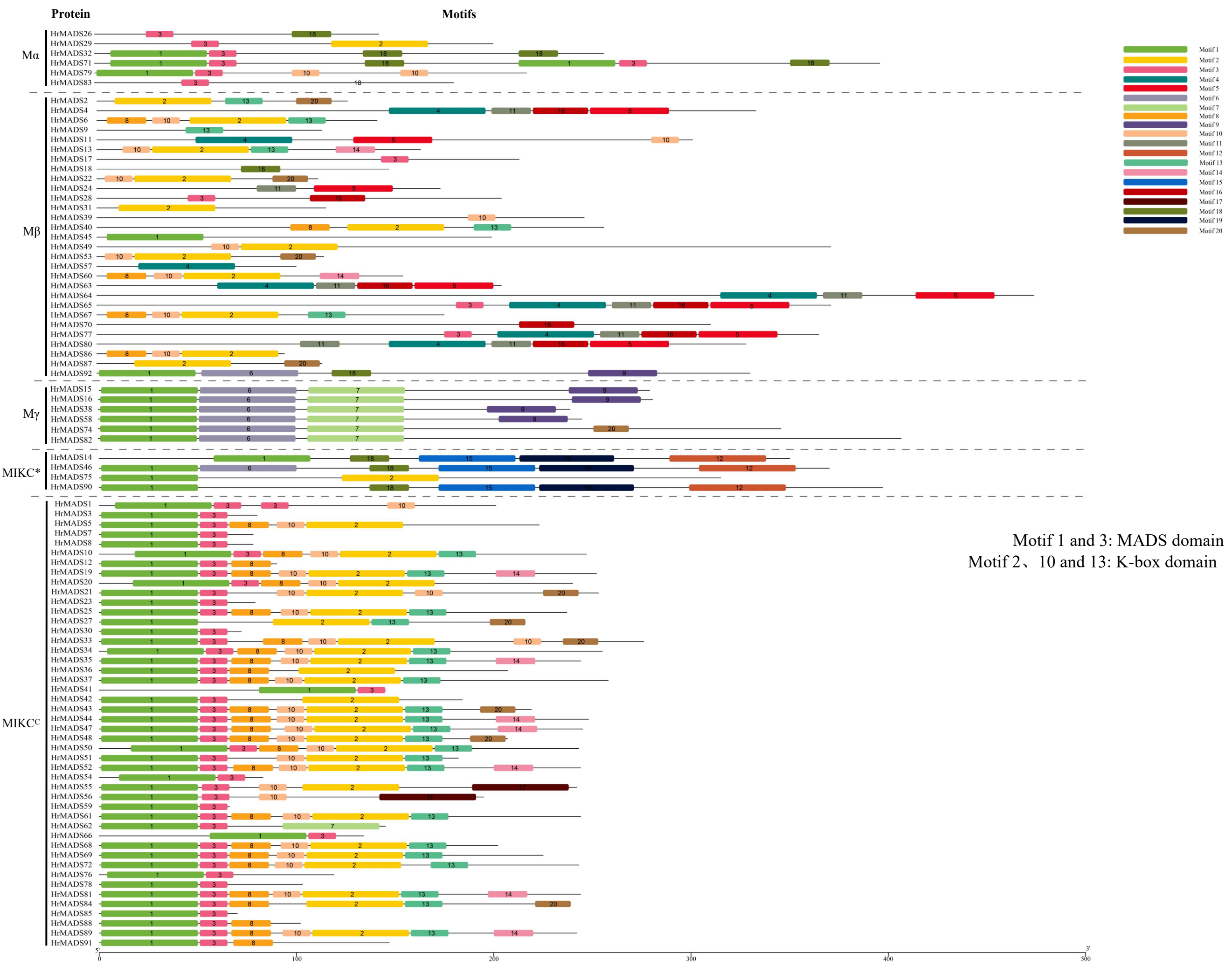

Supplementary Figure 3. Conserved structural domains of the *HrMADS* genes. Different motifs are represented by different colors, with detailed information on each domain in Supplementary Table S2.

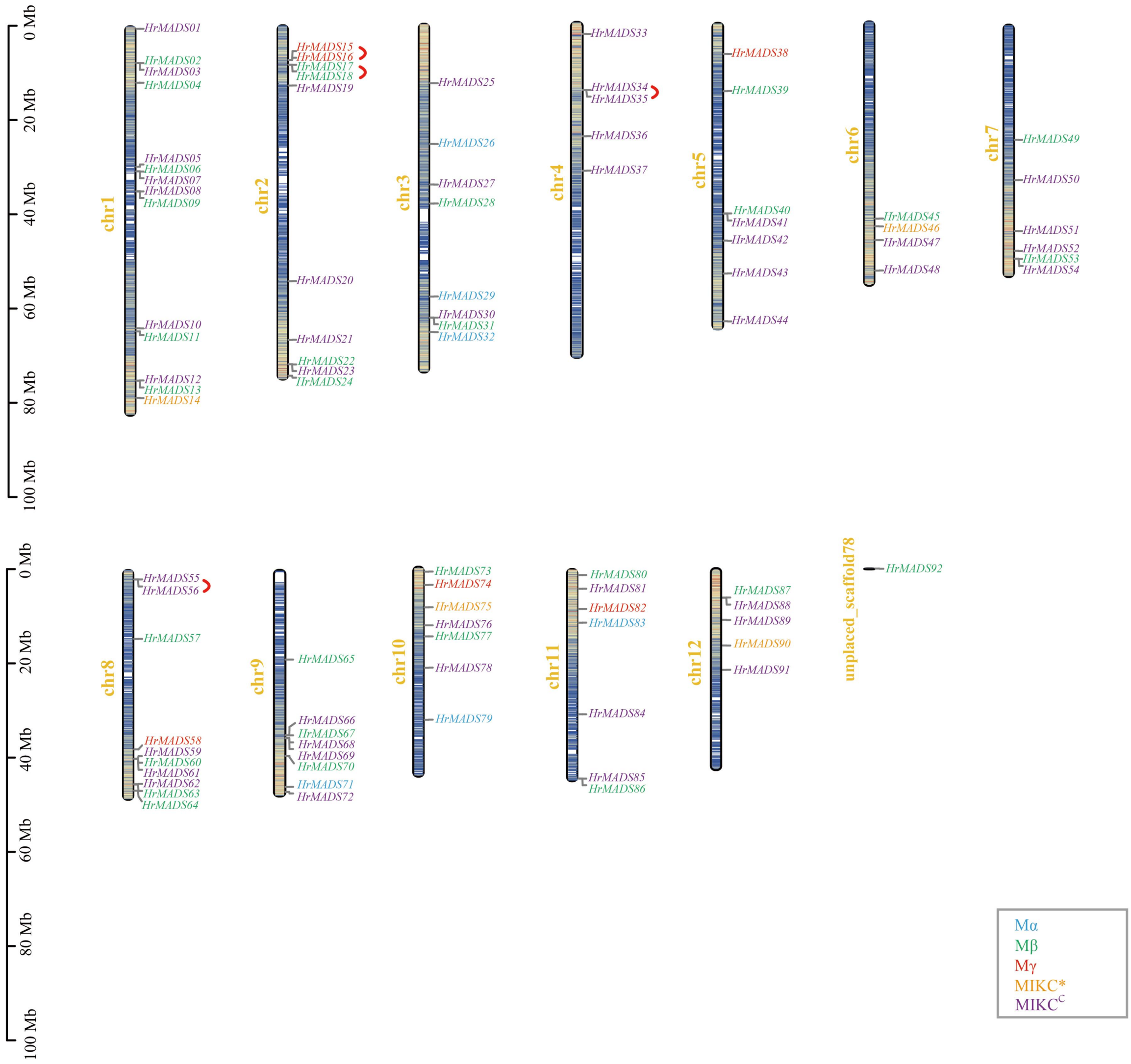

Supplementary Figure 4. Chromosomal distribution of the *HrMADS* genes. A total of 92 *HrMADS* genes are located on 12 chromosomes and 1 scaffold, and the color from blue to yellow on each chromosome indicates gene density. Genes from different subfamilies are shown in different colors. The red linkage represents tandem duplication.

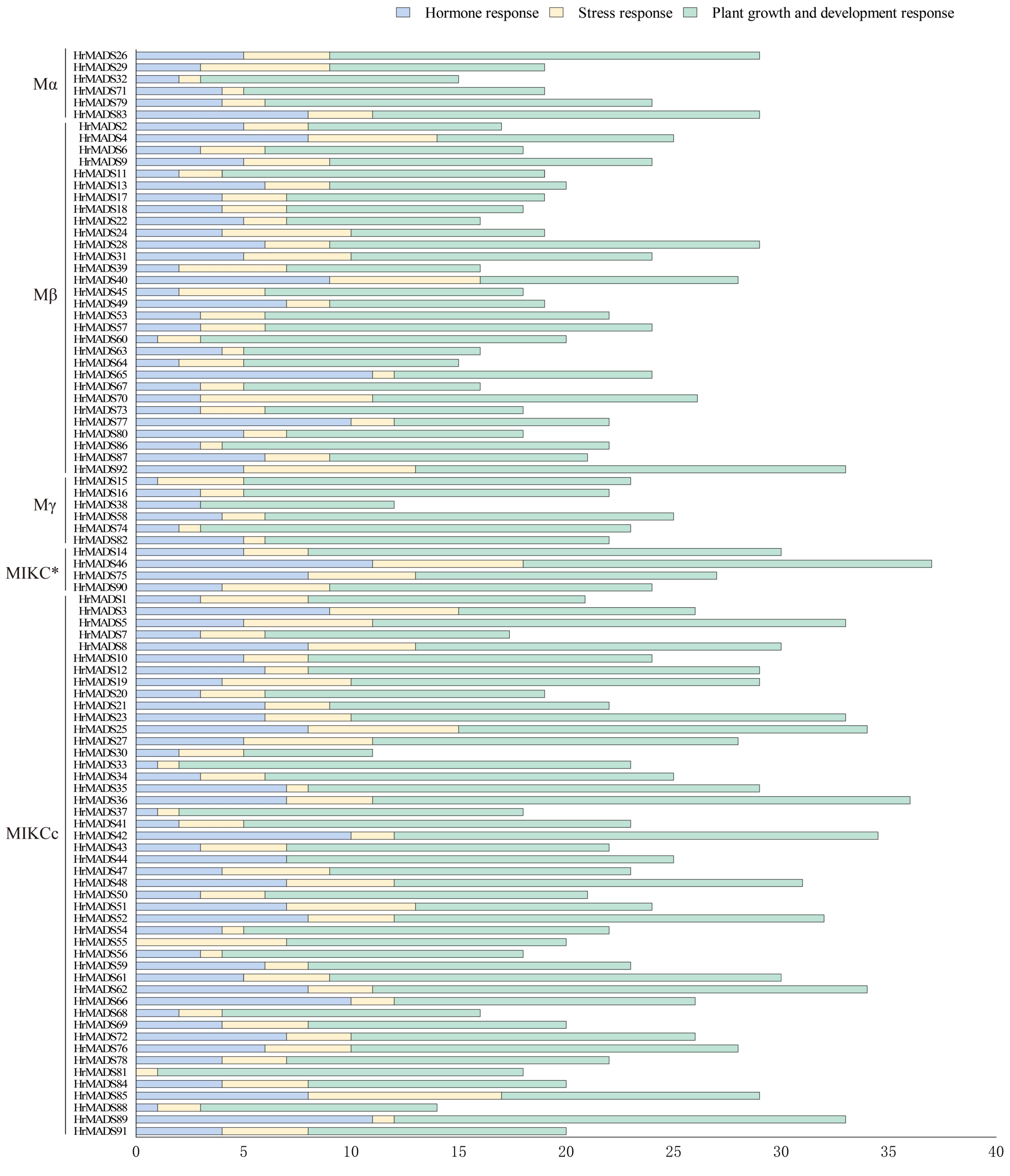

Supplementary Figure 5. Number of cis-acting regulatory elements of the *HrMADS* gene in *H. rhamnoides* ssp. *sinensis*. Different color blocks represent different types of cis-acting elements. Detailed information for each CAE is listed in Supplementary Table S4 and S5.
